# Machine Learning a Predictive Tool for the Analysis of NiCo_2_S_4_/Graphene Composites for Supercapacitor

**DOI:** 10.1002/cssc.202402559

**Published:** 2025-05-27

**Authors:** Heena S. Mulla, Digambar S. Sawant, Sandesh V. Gaikwad, Akash V. Fulari, Rajesh K. Nimat, Deepak P. Dubal, Gaurav M. Lohar

**Affiliations:** ^1^ Department of Physics Lal Bahadur Shastri College of Arts, Science and Commerce Satara 415002 Maharashtra India; ^2^ Department of Physics Balasaheb Desai College Patan Satara 415206 Maharashtra India; ^3^ Symbiosis Centre for Nanoscience and Nanotechnology Symbiosis International (Deemed University) Pune 412115 Maharashtra India; ^4^ School of Chemistry & Physics Centre for Material Science Queensland University of Technology Brisbane Queensland 4000 Australia

**Keywords:** graphene composite, machine learning, nickel‐cobalt sulfide, supercapacitor

## Abstract

Nickel cobalt sulfide (NiCo_2_S_4_) has considerable potential electrode material for supercapacitors owing to its distinct physical and chemical characteristics. However, the practical applications of pristine NiCo_2_S_4_ have been limited by issues such as small specific surface area, agglomeration, and volume changes during cycling, leading to low specific capacitance/capacity and cyclic stability at high rates. Several efforts have been taken to address these challenges. Among those the design and development of NiCo_2_S_4_–graphene‐based composites have been widely investigated. This review explores the effect of NiCo_2_S_4_ architecture and its nanocomposite with graphene on the electrochemical properties. How the various preparative parameters such as synthesis methods, precursors, experimental conditions contributed to efficiently accelerating charge transport kinetics is outlined. Finally, the effect of introduction of graphene on the electrochemical performance of NiCo_2_S_4_ is discussed using density functional theory (DFT). Also, machine learning (ML) models are used to analyze the specific capacitance variation with respect to different synthesis parameters, morphology, energy density, and power density. ML models identify limitations and scope of work for working on NiCo_2_S_4_/graphene composites. It is found that most influencing parameter is annealing time that can alter specific capacitance. The review outlines future research directions, challenges, and opportunities in NiCo_2_S_4_/graphene‐based supercapacitor.

## Graphical abstract

The present review explores the recent advancements of NiCo_2_S_4_/graphene nanocomposite as a promising active material for supercapacitors. Machine learning models are demonstrated to analyze the specific capacitance variation with respect to different synthesis parameters, morphology, energy density, and power density.

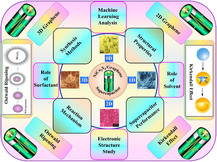

## 1 Introduction

The progress of human development is contributing to a growing global demand for energy.^[^ ^1^, ^2^ ^]^ The role of energy storage systems has become increasingly important in recent years.^[^ ^3^, ^4^ ^]^ A choice of energy storage systems depends on the performance, energy density, charge and discharge capacity, power condition system, safety, and cost.^[^ ^5^, ^6^ ^]^ Batteries such as lead acid,^[^ ^7^ ^]^ nickel hydride, sodium‐sulfur, and lithium‐ion are used as energy storage systems for a miscellaneous purpose.^[^ ^8^, ^9^ ^]^ Every form of battery energy storage system experiences a lifespan limitation, as they all deteriorate and ultimately cease to function over time.^[^ ^10^, ^11^ ^]^ Despite their widespread use, battery technologies have inherent drawbacks, including weight, size, internal resistance (IR), power density, and transient response.^[^ ^12^, ^13^ ^]^ To address these limitations, researchers have investigated supercapacitors as an alternative energy storage device, as they offer promising benefits in comparison to other energy storage devices. Supercapacitors are high‐performance energy storage devices with extremely high specific power and capacitance, capable of rapid charge and discharge cycles. Unlike traditional batteries, they exhibit minimal deterioration over time, making them ideal for applications requiring quick bursts of power and long‐term reliability.^[^ ^14^, ^15^, ^16^, ^17^ ^]^ Two methods of storing charge take place in supercapacitors: one involves a faradic charge transfer mechanism, pioneered by Trasatti et al.^[^ ^18^ ^]^ which utilizes electroactive materials such as transition metal hydroxides/oxides and their combinations; the other involves a non‐faradic charge storage accumulation process, which is well‐described by the Gouy–Chapman–Stern model.^[^ ^19^ ^]^ The practical application‐based supercapacitors devices are categorized into three main types: symmetric, asymmetric, and hybrid, distinguished by the electrode configuration within the supercapacitor cells. Symmetric supercapacitors employ identical electrodes made from the same material, offering balanced performance in terms of charge and discharge storage mechanism.^[^ ^20^, ^21^ ^]^ Asymmetric, on the other hand, utilizes electrodes with different materials or properties, enabling optimized energy density and power density trade‐offs.^[^ ^22^, ^23^, ^24^ ^]^ Hybrid combines features of symmetric and asymmetric designs, often incorporating a combination of traditional carbon‐based electrodes and pseudocapacitive materials, achieving a balance between energy storage capacity and power delivery efficiency.^[^ ^25^, ^26^ ^]^ Each type of supercapacitor configuration serves specific applications, with varying emphasis on factors such as power output, energy density, and cost‐effectiveness. Also, machine learning (ML) is a subfield of artificial intelligence, has emerged as a transformative tool in materials science, offering a data‐driven approach to material discovery and optimization.^[^ ^27^ ^]^ Traditional experimental methods often depend on time‐consuming trial‐and‐error processes, limiting the speed and efficiency of materials development. In contrast, ML controls powerful algorithms to analyze complex datasets, uncover hidden patterns, and predict material properties with remarkable accuracy. This data‐centric approach not only enhances the understanding of material behaviors but also facilitates the identification and design of advanced materials with tailored functionalities. By enabling the efficient exploration of vast parameter spaces, ML is accelerating innovation in various domains of materials science from energy storage and catalysis to electronic and structural materials.^[^ ^28^, ^29^ ^]^

Transition metal sulfides (TMSs) have recently been explored as electrode materials in supercapacitors, primarily because they can deliver high energy density.^[^ ^30^, ^31^ ^]^ They possess properties such as high specific capacitance, cyclic stability, energy density, and power density, making them promising candidates for supercapacitor applications.^[^ ^32^ ^]^ Bimetallic sulfide material has a greater advantage over a single metal sulfide in terms of oxidation states, high theoretical capacitance, and synergistic effect. Researchers have proven that dopant engineering in nanostructured materials is an effective strategy for enhancing electrochemical performance by regulating electronic structures and creating more active sites.^[^ ^33^, ^34^, ^35^, ^36^, ^37^ ^]^ Introducing Co ions into Ni sulfides can produce composite electrodes with Co–Ni sulfide material, which exhibits complementary advantages and is similarly more electrochemically active.^[^ ^38^, ^39^, ^40^ ^]^ Among different TMSs, ternary metal sulfides (MSs) are expected to achieve higher specific capacity compared to single MSs. This is attributed to the synergistic effects arising from the presence of multiple elements, as observed in compounds like CoNi_2_S_4_,^[^ ^41^ ^]^ CuCo_2_S_4_,^[^ ^42^ ^]^ and NiCo_2_S_4_.^[^ ^43^ ^]^ Literature study has been indicated that Ni–Co–S ternary compounds exhibit superior electrochemical characteristics compared to binary compounds because of their narrower bandgaps and increased availability of reaction sites.^[^ ^44^ ^]^ They reported that NiCo_2_S_4_ showed much lower *E*_g_ (absorption bandgap energy) than Nickel cobalt oxide (NiCo_2_O_4_), which is 2.4 and 3.6 eV so much higher conductivity is expected for the NiCo_2_S_4_ sample.^[^ ^45^ ^]^

Among various MSs, NiCo_2_S_4_ has gained considerable attention for its exceptional electrochemical performance. NiCo_2_S_4_ possesses a unique crystal structure, which provides it with unusual electrochemical properties that make it an ideal candidate for supercapacitor applications.^[^ ^46^, ^47^, ^48^, ^49^ ^]^ One of the key advantages of NiCo_2_S_4_ as a supercapacitor material is its high specific capacitance. The unique crystal structure of NiCo_2_S_4_ facilitates efficient charge storage at the electrode–electrolyte interface, leading to a high capacitive behavior.^[^ ^39^, ^50^ ^]^ This high specific capacitance enables the supercapacitor to store a larger amount of electrical energy per unit mass, thereby enhancing its energy storage capacity. Moreover, NiCo_2_S_4_ exhibits excellent electrical conductivity, which is crucial for efficient charge transfer during the electrochemical processes.^[^ ^33^, ^47^, ^51^ ^]^ Because of the inherent limitations of TMSs characterized by low electronic conductivity during redox reactions, the utilization of NiCo_2_S_4_ as battery‐type electrode material in supercapacitors results in unsatisfactory electrochemical reversibility during cyclic stability.^[^ ^52^, ^53^ ^]^ Several studies have consistently shown that metallic sulfides tend to have lower electron conductivity. To address this limitation, the researcher explored the possibility of enhancing the electron conductivity of MSs by incorporating carbon nanofillers, such as carbon nanotubes,^[^ ^54^ ^]^ reduced graphene oxide (rGO),^[^ ^55^ ^]^ and 3D graphene.^[^ ^56^, ^57^ ^]^ By incorporating graphene or other materials, researchers enhanced the electron conductivity of these MSs, forming hierarchical structures that improved the performance of supercapacitors.^[^ ^58^, ^59^, ^60^ ^]^ The integration of NiCo_2_S_4_ and rGO in a composite structure combines their respective advantages, resulting in synergistic effects for superior supercapacitor performance. The incorporation of rGO into the NiCo_2_S_4_ electrode promotes electron transport, reduces IR, mechanical strength, and improved conductivity.^[^ ^56^, ^61^ ^]^ These effects contribute to enhanced charge storage capacity, reduced charge transfer resistance, and improved overall electrochemical performance of the supercapacitor. The NiCo_2_S_4_‐rGO hybrid (HSC) exhibits impressive specific capacitance, enabling it to store a larger amount of electrical energy per unit mass. The combination of NiCo_2_S_4_ and graphene similarly leads to excellent rate capability, enabling rapid charge/discharge rates without significant performance degradation. Understanding the specific interactions and bonding between NiCo_2_S_4_ and rGO is essential for optimizing the performance of the composite material in distinct potential applications, such as energy storage devices and catalysis.^[^ ^58^, ^62^, ^63^, ^64^ ^]^

Several review articles have been published on NiCo_2_S_4_ for energy storage applications. Yi et al.^[^ ^65^ ^]^ explore recent advances in NiCo_2_S_4_ composites for supercapacitors, batteries, and their synthesis methods. They investigated how nanostructures and composites with functional materials influence the electrochemical properties of NiCo_2_S_4_‐based materials. Xue et al.^[^ ^66^ ^]^ provided the contribution of NiCo_2_S_4_ for supercapacitors and explored how its structure, developing methods, and underlying function impact its energy storage capabilities. Zhao et al.^[^ ^67^ ^]^ give information regarding recent progress and modification strategies of NiCo_2_S_4_ for electrochemical properties and energy storage mechanism of NiCo_2_S_4_ discussed. Besides, it did not provide any information about the impact of surfactant. In a previous review, they did not consider theoretical aspect and theoretical calculation about an electronic structure and bandgap. However, the progress in NiCo_2_S_4_‐graphene based composite for supercapacitors has never been systematically summarized considering reaction mechanism, synthesis methods, and experimental conditions.

The present review article fills the gap by providing a comprehensive summary on the synthesis methods, NiCo_2_S_4_/graphene composite, a growth mechanism of NiCo_2_S_4_ on graphene with different sulfur precursors, and their electrochemical properties. Similarly, we summarized the growth mechanism and sulfurization process with the help of the Ostwald ripening and Kirkendall effect. Here, we differentiate zero dimensional (0D), one dimensional (1D), two dimensional (2D), and three dimensional (3D) morphological nanostructures of NiCo_2_S_4_/graphene composites like graphene oxide (GO) and rGO for their electrochemical properties as well as studied the effect of 2D and 3D graphene nanostructures on electrochemical performance. 3D graphene is a new engineered architecture introduced in graphene‐based nanocomposite, which is discussed in our review. We studied the role of surfactant/stabilizer on a growth mechanism and nanostructure development concerning sodium dodecyl sulfate, NH_4_F, and polyvinyl pyrrolidone. We comprehensively studied the role of the solvent and its effects on the nucleation, growth rate, and morphology of nanostructured materials. Theoretical investigation of the effect of graphene on the electrochemical performance of NiCo_2_S_4_ is discussed with the help of density functional theory in this review. Density functional theory (DFT) involves a local density approximation (LDA), generalized gradient approximation (GGA) under PW91, and Perdew, Becke, and Ernzerhof (PBE). This review explores the first‐ever application of ML of NiCo_2_S_4_/graphene composite and its device performance to analyze the material properties with different models. In ML, we have studied the effect of experimental conditions, different nanostructures, and surface area on specific capacitance. Also, we have studied fabricated devices and analyze the correlation between energy density, power density, and cyclic stability concerning specific capacitance from the ML approach. After extensive literature survey, we never found any article explained NiCo_2_S_4_/graphene supercapacitor analysis on the basis of experimental parameters and its behavior prediction.

## 2 Method of Synthesis

Generally, the synthesis of MSs follows the dissolution‐recrystallization mechanism, commonly known as the “oxide‐to‐sulfides” strategy.^[^ ^68^ ^]^ Modifying the morphologies, structures, sizes, and compositions of MSs allows for the fine adjustment of their electrochemical characteristics. Therefore, the selection of synthesis methods plays a crucial role in tailoring MSs with desired properties, ultimately enhancing the energy storage properties of the material. Researchers have employed assorted synthesis techniques for MSs including hydrothermal/solvothermal methods,^[^ ^69^, ^70^ ^]^ precipitation,^[^ ^71^ ^]^ electrodeposition,^[^ ^72^ ^]^ microwave‐assisted synthesis,^[^ ^73^ ^]^ and chemical vapor deposition.^[^ ^74^ ^]^ NiCo_2_S_4_ is synthesized by different methods that include hydrothermal method, one pot refluxing method,^[^ ^75^ ^]^ one‐pot method,^[^ ^76^ ^]^ solvothermal,^[^ ^77^ ^]^ electrodeposition,^[^ ^78^ ^]^ chemical bath deposition,^[^ ^79^ ^]^ etc. Over the past few decades, there has been significant research into the synthesis of hollow micro and nanostructures ranging in size from 10 nm to 10 μm, driven by their potential applications. Diverse synthesis processes, including Ostwald ripening,^[^ ^80^, ^81^, ^82^ ^]^ layer‐by‐layer (LBL) assembly,^[^ ^83^, ^84^ ^]^ and the Kirkendall effect,^[^ ^85^, ^86^ ^]^ have been employed to create micro/nanomaterials with diverse nanostructures such as core–shell and hollow configurations. Among these processes, the Kirkendall effect has garnered particular attention for shaping engineered hollow nano‐microstructures of metal oxides and sulfides due to its promising applications.^[^ ^87^, ^88^ ^]^

The Ostwald ripening process is commonly observed in varied synthesis methods, but it is particularly prominent in the synthesis of nanoparticles through solution‐phase methods such as chemical precipitation,^[^ ^89^ ^]^ solvothermal,^[^ ^90^ ^]^ and hydrothermal methods.^[^ ^91^ ^]^ In these methods, nanoparticles initially form as small primary particles, which then undergo Ostwald ripening where smaller particles dissolve and their material is redeposited onto larger particles, causing them to grow over time as shown in Graphical Fig. This process occurs due to differences in surface energy, where smaller particles have higher surface energy than larger ones. This phenomenon leads to a gradual increase in the average size of nanoparticles and a more uniform size distribution. This occurs due to differences in solubility or chemical potential between smaller and larger particles. Therefore, Ostwald ripening is frequently utilized and observed in nanoparticle synthesis methods involving solution‐phase reactions.^[^ ^92^ ^]^ Since the initial development of hollow nanocrystals using the nanoscale Kirkendall effect, significant progress has been made in utilizing this approach to create hollow nanostructures in recent years. The nanoscale Kirkendall effect, which relies on mass transport across the interface between different solid phases, has been effectively employed to synthesize hollow nanocrystals spanning several materials categories such as metal oxides,^[^ ^93^ ^]^ chalcogenides,^[^ ^94^ ^]^ phosphides,^[^ ^95^ ^]^ sulfides,^[^ ^96^ ^]^ as well as core/hollow shell structures.^[^ ^97^, ^98^ ^]^ The Kirkendall effects are observed during the formation of hollow particles of NiCo_2_S_4_ as shown in Graphical Fig.^[^ ^99^ ^]^ The experimental study suggests that metal cations diffuse more rapidly than sulfide anions, potentially due to the smaller size of outward‐diffusing metal cations compared to inward‐diffusing sulfide anions. This observation aligns with the mechanism of the Kirkendall effect. The Kirkendall effect, observed in diffusion between materials with differing atomic mobilities, results in uneven interface growth and the formation of voids or pores in the slower‐diffusing material. This phenomenon is widely utilized in materials science to engineer unique nanostructures, such as NiCo_2_S_4_ nanotubes formed via sacrificial templating. The faster diffusion of the inner Ni–Co precursor compared to S^2−^ ions during vulcanization leads to void generation, resulting in porous tube nanostructures. These nanotubes exhibit exceptional electrochemical performance, attributed to active sites facilitating redox transitions at the electrode surface.^[^ ^100^, ^101^ ^]^ In the formation process of NiCo_2_S_4_, the Kirkendall effect induces the formation of a hollow and mesoporous structure, which offers multiple advantages for electrochemical performance. First, the hollow structure provides a larger surface area^[^ ^102^ ^]^ than a solid counterpart, facilitating increased contact between the electrode and electrolyte, thereby promoting more efficient electrochemical reactions. Second, the presence of mesopores enhances ion transport within the electrode material, mitigating diffusion limitations and enabling rapid charge and discharge rates. Lastly, the hollow architecture aids in alleviating volume changes during cycling, reducing mechanical stress, and improving the electrode's long‐term structural stability.^[^ ^103^, ^104^ ^]^ Zhu et al.^[^ ^105^ ^]^ formulated Ni–Co carbonate and then transformed it into NiCo_2_S_4_. The structure of nickel cobalt (carbonate) hydroxide can be effectively managed via solvothermal reactions, and NiCo_2_S_4_ can be transformed from nickel cobalt (carbonate) hydroxide (for example, NiCo_2_((CO_3_)_1.5_(OH)_3_) through anion exchange reactions induced by Kirkendall effects.^[^ ^62^ ^]^

### 2.1 Hydrothermal Method

Researchers have recognized the utilization of hydrothermal conditions for processing fine particles.^[^ ^106^ ^]^ Hydrothermal is a widely used method for the synthesis of nanostructure via bottom‐up approach because of its significance such as single step, low energy consumption, eco‐friendly, low cost, highly pure product, and different morphologies. Studies showed that the hydrothermal method is widely used for the synthesis of sulfide because of its significant benefits.^[^ ^107^, ^108^, ^109^, ^110^ ^]^ This method excels in refining ultrafine powders, exact stoichiometry, uniformity, dense particle structure, heightened crystallinity, increased reactivity, and enhanced sinterability, among numerous other advantages.^[^ ^111^, ^112^ ^]^ Yang et al.^[^ ^113^ ^]^ prepared NiCo_2_S_4_ via a hydrothermal method that includes three steps. Which involved the synthesis of NiCo_2_O_4_, and it was converted into NiCo_2_S_4_ via sulfurization. The precursors of cobalt and nickel form Co^2+^ and Ni^2+^ ions in an aqueous medium, these cations reacted with urea (Co_3_^2−^ and OH^−^) hydrolyzing agent to form a pink colored bimetallic hydroxide carbonate, which grew on nickel foam (NF). After that, the previous nickel–cobalt hydroxide carbonate was converted into NiCo_2_O_4_ through the annealing process (300 °C). In the third step, obtained NiCo_2_O_4_ was converted into NiCo_2_S_4_ via an anion exchange process.

### 2.2 Microwave‐Assisted Method

Microwave heating typically induces internal heat within molecules through dielectric polarization. This heating method offers multiple advantages, such as being noncontact, selective, and rapid.^[^ ^114^ ^]^ The microwave technique proves to be a valuable strategy for mitigating certain limitations, such as swift heating, rapid reaction rates, elevated product yields, exceptional reproducibility, extended reaction conditions, and efficient energy transformation.^[^ ^115^, ^116^ ^]^ Recently, NiCo_2_S_4_ was prepared through the microwave‐assisted method by Rafai et al.^[^ ^117^ ^]^ and it is a short‐time method as compared to another method. Likewise, Wang et al.^[^ ^118^ ^]^ fabricated NiCo_2_S_4_ nanosheet arrays grown on NF via microwave‐assisted method. Microwave‐assisted heating was acknowledged as a method for crafting or thermally treating conventional functional materials. The dipolar polarization and ionic conduction heating mechanisms allowed for a significant reduction in the reaction time of microwave‐assisted processes to a matter of within a short time. Zhao et al.^[^ ^119^ ^]^ produced NiCo_2_S_4_ via the microwave‐assisted method and controlled the growth of the structure by varying in time. They developed nanoparticles to hydrangea‐like NCs through the time‐controlled microwave‐assisted method. Due to the swift internal heating mechanism, a substantial quantity of monomer nuclei and crystals can be rapidly generated. These entities then promptly mature into nanosheets through the crystallization process. Over time, as the growth‐controlled process shifts toward thermodynamic dominance, the nanosheets progressively undergo self‐assembly, forming a 3D hydrangea‐like structure. This structural arrangement aims to minimize the surface energy of the nanosheets.

### 2.3 Electrodeposition Method

Electrochemical deposition is commonly employed for the direct growth of metals and conducting metal oxides due to its versatility in manipulation and its lack of need for high vacuum or high reaction temperatures.^[^ ^120^, ^121^, ^122^ ^]^ A simple and cost‐effective single‐step electrodeposition technique was employed to produce NiCo_2_S_4_ nanosheets with varying compositions.^[^ ^123^ ^]^ Tauquir and co‐workers^[^ ^124^ ^]^ prepared a NiCo_2_S_4_ nanosheet by facile in situ growth via the electrodeposition method. The experiment involved different concentrations of nickel and cobalt nitrate hexahydrate with 0.75 m of thiourea and 50 mm ascorbic acid. The electrodeposition took place using chronoamperometry techniques for 10 min at −1.2 V. While for cyclic voltammetry (CV) deposition the potential was kept between −1.2 and 0.2 V at a scan rate of 10 mV s^−1^ for 10 cycles.

### 2.4 Successive Ionic Layer Adsorption and Reaction Method

The successive ionic layer adsorption and reaction (SILAR) method was employed to synthesize nanomaterials for SC applications. In addition to being low‐cost, simple, and eco‐friendly, the SILAR technique was more economically stable and allowed for more applications compared to other physical and chemical methods. SILAR offers a unique approach to producing nanocrystalline films.^[^ ^125^ ^]^ Shinde and coworkers^[^ ^126^ ^]^ studied the influence of the SILAR cycle on the SC performance of nanoflakes, nanosheets, and nanoplates like NiCo_2_S_4_ electrodes. The synthesis process involved three steps using the SILAR method. In the initial step, a flexible stainless‐steel substrate was immersed in NiSO_4_ solution for 20 s, facilitating the adsorption of Ni^2+^ ions on the substrate's surface. Subsequently, the Ni^2+^‐coated substrate was rinsed with double distilled water (DDW) for 10 s to eliminate loosely bound Ni^2+^ species. In the second step, the Ni^2+^‐coated substrate underwent a 20‐second immersion in CoSO_4_ solution, allowing the adsorption of Co^2+^ ions. A similar process was employed to remove loosely bound Co^2+^ ions. Finally, in the third step, the Ni^2+^/Co^2+^‐coated substrate was immersed in Na_2_S precursor solution, forming a Ni^2+^‐, Co^2+^‐, and S^2−^‐coated substrate. A 10 s rinse with DDW removed roughly bound S^2−^ ion species, completing one SILAR cycle of NiCo_2_S_4_ deposition.

### 2.5 Simple Chemical Method

**Figure 1:**
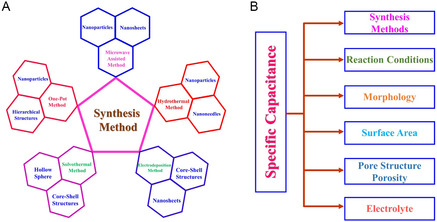
A) Correlation between synthesis method and observed morphology. B) Parameters influencing specific capacitance.

The one‐step simple chemical method is simple and facilitated by rational construction. This results in the creation of distinctive hierarchical nanostructures that offer reduced transferring resistance, a larger surface area, and improved structural stability. These attributes are advantageous for facilitating electrolyte ion diffusion and establishing more efficient pathways for electron transport. A one‐step synthesis method combines multiple processes into a single step, enhancing interactions between components and leading to a synergistic effect. This contrasts with simple physical mixing, which may not promote the same level of interaction and synergy.^[^ ^56^, ^127^ ^]^ Wang et al.^[^ ^128^ ^]^ developed a simple one‐step temperature solution‐based synthesis approach for the fabrication of hierarchical NiCo_2_S_4_ ultrathin nanosheets on NF. The hydrolyzation process of thiourea tends to be a solution to alkaline resulting in the generation of alkaline medium (ammonia) and sulfur source (S^2−^). A low reaction temperature (90 °C) leads to a slow hydrolyzation rate, which is helpful in the formation of ultrafine nanostructures. Also, chemical coprecipitation and sulfidation are two strategies combined into a single step, which favors time‐saving and low‐cost methods. Also, the mass loading of NiCo_2_S_4_ was controlled by tuning a reaction time.^[^ ^129^ ^]^ At a time, soluble ions of Ni^2+^ and Co^2+^ were coordinated and form a Ni–Co hydroxide on NF in alkaline conditions, thoroughly the prepared hydroxide was converted into a NiCo_2_S_4_ by reacting with S^2−^ via an ion exchange process through a one‐step method.^[^ ^130^ ^]^ **Figure** **1**A illustrates the commonly used synthesis method for NiCo_2_S_4_/graphene composite, highlighting the correlation between the synthesis method and the observed morphology. Figure 1B presents the key parameters influencing specific capacitance. The morphology of the NiCo_2_S_4_/graphene composite is primarily determined by the synthesis method and reaction conditions. By modifying these factors, a desirable morphology can be achieved. To evaluate the electrochemical performance of NiCo_2_S_4_/graphene composite, specific capacitance parameters are analyzed, as they are influenced by morphology, surface area, pore structure, and electrolyte composition.

## 3 Structural Properties of NiCo_2_S_4_

It is crucial to emphasize that chalcogenide spinel, along with their oxide counterparts, represents the most extensive category of inorganic compounds,^[^ ^131^ ^]^ However, the characteristics of transition metal chalcogenides markedly differ from those of oxides due to the involvement of “d” electrons in covalent bonding within chalcogenides characterized by closely packed anion sublattices.^[^ ^132^ ^]^ A top‐down ion exchange process to sulfurize an epitaxial NiCo_2_O_4_ film, resulting in the transformation of the original semiconductor NiCo_2_O_4_ film into a metallic NiCo_2_S_4_ film.^[^ ^133^ ^]^ Following sulfurization, there was a switch in the carrier type from p‐type to n‐type. Magnetic properties undergo alterations NiCo_2_O_4_ exhibits ferromagnetism,^[^ ^134^ ^]^ whereas NiCo_2_S_4_ displays paramagnetism,^[^ ^135^ ^]^ suggesting that the magnetic state of NiCo_2_S_4_ can be manipulated through oxidation. Magnetic and electrical characteristics of NiCo_2_S_4_ samples subjected to varying degrees of oxidation. An octahedral distortion was observed in the lattice of partially oxidized NiCo_2_S_4_, resulting in the transformation of Co^3+^ ions from a low spin to an intermediate spin state. These alterations in the spin and valence states of the metal atoms consequently led to changes in the magnetic state of the oxidized NiCo_2_S_4_ powders. Notably, a transition from an antiferromagnetic phase to a ferromagnetic phase was identified in the partially oxidized NiCo_2_S_4_ powders.^[^ ^136^ ^]^ NiCo_2_S_4_ typically adopts a spinel crystal structure, characterized by the arrangement of Ni and Co cations in octahedral and tetrahedral sites within the cubic close‐packed sulfur anions. Its crystal structure belongs to a specific Fd‐3*m* space group, governing its symmetry and lattice parameters.^[^ ^137^ ^]^ Crystals can have different phases depending on factors such as temperature, pressure, and composition. The heat treatment on precursor diffraction pattern initially corresponds to the spinel NiCo_2_O_4_ phase with Fd‐3*m* symmetry, but after sulfuration treatment, it indexes well with diffraction peaks corresponding to the cubic NiCo_2_S_4_ phase.^[^ ^138^ ^]^ The crystal structure of NiCo_2_S_4_, and in a crystal structure, Ni and Co are situated in tetrahedral and octahedral positions, respectively. In the unit cell of NiCo_2_S_4_, half of the octahedral positions are occupied by Co^3+^, while Ni^2+^ occupies one‐eighth of the tetrahedral positions.^[^ ^65^ ^]^ NiCo_2_S_4_ and NiCo_2_O_4_ both belong to the Fd‐3*m* space group. When comparing NiCo_2_S_4_ to its respective oxide, NiCo_2_O_4_, it is observed that NiCo_2_S_4_ has a larger lattice constant of 9.387 Å, while NiCo_2_O_4_ has a lattice constant of 8.114 Å. This difference is attributed to the variance in ionic radii between sulfur and oxygen. The ionic radius of S^2−^ is 184 pm, which is greater than the ionic radius of O^2−^ at 140 pm. This variance in ionic radii contributes to the disparity in lattice constants between NiCo_2_S_4_ and NiCo_2_O_4_.^[^ ^139^, ^140^ ^]^ With the increasing substitution of oxygen (O) for sulfur (S) atoms, the degree of overlap with the metal ion orbitals increases, and the external 3*d* electrons of metal ions become localized.^[^ ^141^ ^]^ Knop et al.^[^ ^142^ ^]^ employed neutron diffraction to unveil the crystal structure of (Ni)A[Co_2_]BS_4_, confirming it to be a classic spinel structure. The atoms within the material are arranged in a specific, three‐dimensional pattern characteristic of the spinel. Nickel (Ni) occupies A sites within the crystal lattice, while cobalt (Co) atoms reside in specific B sites, both surrounded by sulfur (S) atoms. This distinct arrangement, confirmed through neutron diffraction analysis, holds the key to understanding the material's properties and potential applications. The spinel‐type crystal structure of AB_2_S_4_, where A and B can represent elements such as Ni, Co, Fe, Cu, and others, features metal A cations occupying the tetrahedral sites and metal B cations occupying the octahedral sites.^[^ ^143^ ^]^ Research has demonstrated that nickel and cobalt are situated at the tetrahedral and octahedral sites, respectively, within this compound. This structural arrangement contributes to the unique properties and potential applications of spinel NiCo_2_S_4_ in energy storage technologies.^[^ ^144^, ^145^ ^]^

## 4 Role of Surfactant/Stabilizer in Formation of NiCo_2_S_4_ Nanostructures

The role of surfactants in the synthesis of nanostructured materials is crucial as they can influence the morphology and properties of the final product. In the study on the synthesis of NiCo_2_S_4_ nanostructures, the surfactant plays a significant role in determining the morphology of the obtained structures. The choice of surfactants can influence the growth mechanism, crystal structure, and morphology of the synthesized nanostructures. Surfactants can interact with the metal ions and other reagents in the solution, leading to the formation of specific nanostructures.^[^ ^146^, ^147^ ^]^ Sodium dodecyl surfactant, NH_4_F, and polyvinyl pyrrolidone are generally used as surfactant or stabilizers. Surfactants act as structure‐directing agents in the hydrothermal synthesis process.

### 4.1 Sodium Dodecyl Sulfate

In the hydrothermal synthesis process, Ni^2+^ and Co^2+^ ions in an aqueous solution react with C_2_H_5_N·S‐SDS ions to produce cubohexa‐octahedral NiCo_2_S_4_ nanostructures. The anionic sodium dodecyl sulfate (SDS) plays a crucial role by disrupting noncovalent and electrostatic bonds during nucleation, while also enhancing interactions on both polar and nonpolar surfaces. C_2_H_5_N·S‐SDS serves as a reservoir for S^−^ ions. The formation of unique NCS‐COH nanostructures is attributed to several factors: the hydrothermal reaction system providing initial nuclei, SDS guiding the formation of quadrangles and cubical hexagons with a high surface area, and the gradual vulcanization process aiding in morphology shrinkage. Moreover, during ion exchange, CO_3_^2−^and OH^−^ ions may replace S^2−^ ions, with CO_3_^2−^ and OH^−^ reacting with H^+^ ions from the hydrolysis of C_2_H_5_N·S‐SDS to produce CO_2_ and H_2_O, thereby stabilizing the original structure. The combination of these mechanisms results in the distinct construction of NiCo_2_S_4_ nanostructures.^[^ ^148^ ^]^ NiCo_2_S_4_ pure precursor exhibits agglomerate nanoneedles with an average diameter of ≈100 nm and lengths reaching 1–2 μm. In contrast, the NiCo_2_S_4_‐SDS precursor features self‐organized nanoneedles with sharp tips, boasting an average diameter of 80 nm and similar lengths. The presence of SDS serves as an effective anionic surfactant, preventing agglomeration of NiCo_2_S_4_ nanoparticles. SDS hydrophilic terminal absorbs Ni^2+^ and Co^2+^ ions, facilitating direct nucleation and growth along the SDS chains in a hydrothermal setting. By growing NiCo_2_S_4_‐SDS nanostructured electrodes on NF, strong mechanical adhesion and electrical contact are ensured without the need for binders or conducting additives. The role of SDS in a hydrothermal environment significantly impacts the crystal structure, morphology, and electrochemical properties of NiCo_2_S_4_ electrodes.^[^ ^149^ ^]^

### 4.2 Ammonium Fluoride (NH_4_F)

The concentration of ammonium fluoride (NH_4_F) plays a crucial role in synthesizing various morphologies in the synthesis of NiCo_2_S_4_. The growth mechanism suggests that with limited NH_4_F, the NF surface activates as effective sites for crystal nucleus growth, leading to 1D growth. Increasing NH_4_F activates more surface areas, resulting in 2D growth and eventual flakes formation. Further increase in NH_4_F leads to shorter flakes. At a certain level, all surfaces are activated, prompting 3D growth. Excess F^−^ ions etch through the material, yielding 3D. It is also important to ensure a tight connection between the Ni substrate and the base.^[^ ^150^ ^]^ Without NH_4_F, only 1D Ni‐Co precursor nanowire arrays were achieved. However, with NH_4_F at 0.2 m, (Nanosheets@nanowires) arrays were obtained, while at 0.24 m only nanosheet arrays were observed. Increasing the concentration to 0.3 m led to the appearance of hexahedron arrays. The presence of NH_4_F was found to promote the growth of 2D and 3D Ni‐Co precursors on NF, resulting in nanosheets and hexahedron structures. Conversely, without NH_4_F, the growth of 1D nanowires was favored. It was noted that the Ni–Co precursor is unstable in acidic environments, especially for lower‐dimensional nanomaterials with high surface areas. Higher concentrations of NH_4_F favored the formation of 2D or 3D nanostructures.^[^ ^151^ ^]^ The presence or absence of surfactants like NH_4_F can result in the formation of different morphologies, such as nanoneedles, nanosheets, flower‐like structures, and caterpillar‐like arrays. When urea is used as the alkali source, high‐density 1D NiCo_2_S_4_ nanoneedle arrays are grown on NF vertically. With the addition of NH_4_F, these nanoneedles transform into a 3D caterpillar‐like morphology composed of both 1D nanoneedles and 2D nanosheets. The presence of NH_4_F can lead to forming 3D flower‐like structures or closely spaced 2D nanosheets, depending on the surfactant used. The surfactant influences the interactions between the nanostructures, such as avoiding the aggregation of nanosheets or promoting the aggregation of certain morphologies.^[^ ^152^ ^]^

### 4.3 Polyvinyl Pyrrolidone (PVP)

Polyvinyl pyrrolidone (PVP) is likely a stabilizing agent, PVP is a polymer that can form complexes with metal ions, helping to control the size, shape, and dispersion of the nanoparticles during synthesis. It helps to control the size and shape of the nanospheres by preventing the nanoparticles from aggregating.^[^ ^38^ ^]^ The use of PVP‐assisted solvothermal procedure marks a significant advancement in the synthesis of highly uniform and monodisperse solid spheres. By employing this technique, researchers can achieve accurate control over the size and shape of the resulting spheres, leading to enhanced uniformity, and can also influence their morphology and crystallinity. PVP controls the growth of nanospheres as the amount of PVP increases the size of nanospheres decreases.^[^ ^153^ ^]^

## 5 Role of Solvents in the Formation of NiCo_2_S_4_ Nanostructures

Solvent effects play a crucial role in the growth of nanostructured materials as they can significantly influence the formation and morphology of the final product. When a material is synthesized in a solution, the choice of solvent can impact various aspects of the reaction, including nucleation, growth rate, and crystal orientation. The influence of solvent ratios on morphology control and suggesting tailored synthesis for enhanced supercapacitor performance. Different NiCo_2_S_4_ architectures, such as urchin‐like, flower‐like, tube‐like, and cubic‐like structures, were synthesized via a shape‐controlled hydrothermal route using various solvents, such as DW, ethanol, and glycol in different ratios impacting morphology and electrochemical performance.^[^ ^154^, ^155^ ^]^

### 5.1 Ethanol

It served as a solvent, effectively dissolving precursor salts and compounds, enabling the formation of a homogeneous solution conducive to the reaction between metal ions and sulfur sources. Acting as a reaction medium, ethanol facilitated controlled nucleation and growth of NiCo_2_S_4_ nanoparticles during the solvothermal or hydrothermal process, ensuring desired morphology and size distribution. Furthermore, ethanol acted as a stabilizer and surfactant, effectively controlling nanoparticle growth and aggregation, thereby preventing particle agglomeration and promoting the formation of well‐dispersed, uniform nanoparticles. NiCo_2_S_4_ microspheres were effectively synthesized using a template‐free solvent‐thermal technique, utilizing isopropyl alcohol and ethanol as the solvent components.^[^ ^49^, ^156^ ^]^

### 5.2 Glycol

Ethylene glycol is used as a solvent in the solvothermal method,^[^ ^147^, ^157^ ^]^ by utilizing the properties of alcohol viscosity, a combination of isopropanol and triethylene glycol is employed instead of an aqueous solution to regulate the seed growth rate and achieve uniformly structured NiCo_2_S_4_ materials with flaky hollow‐sphere attachments. Serving as an electrode material, the prepared NiCo_2_S_4_ demonstrates remarkable capacitance, outstanding cyclic stability, and high‐rate capability.^[^ ^127^ ^]^ Ethylene glycol also serves as a reducing agent for metal cations. Ethanol and ethylene glycol are commonly used in growth procedures as dispersants and solvents.^[^ ^158^ ^]^

## 6 Morphology‐Based Supercapacitor Performance of NiCo_2_S_4_/Graphene Composite

In graphene, GO and rGO are both 2D carbon materials known for their exceptional properties, including high surface area and excellent electrical conductivity. These characteristics make them highly attractive for applications in supercapacitors. Graphene, a single layer of carbon atoms arranged in a hexagonal lattice, offers phenomenal electron mobility and mechanical strength. The rGO, produced from GO through reduction processes, retains many of graphene's properties while also exhibiting improved conductivity due to the removal of oxygen‐containing functional groups. Both materials provide a large surface area for electrochemical reactions, facilitating the efficient storage and release of electrical energy, thus making them extensively studied for supercapacitor applications.^[^ ^64^, ^159^ ^]^ Graphene and rGO exhibit a planar 2D structure that makes them excellent supports for NiCo_2_S_4_ due to their large surface area and close contact, facilitating effective dispersion and anchoring of NiCo_2_S_4_ nanoparticles. The inherent high electrical conductivity of graphene and rGO enables swift charge transfer during the charge‐storage process, reducing IR and optimizing active material utilization. This synergistic interaction between NiCo_2_S_4_ and graphene (or rGO) enhances the capacitive performance of composites by promoting efficient electrochemical reactions at the electrode/electrolyte interface, ultimately boosting energy storage and delivery capabilities in supercapacitor applications.^[^ ^160^, ^161^ ^]^ The development of 3D NiCo_2_S_4_‐rGO composites with controlled pore size distribution is essential for further enhancing supercapacitor performance.^[^ ^162^ ^]^ Here, the classification of NiCo_2_S_4_ is elaborated based on morphology, highlighting graphene as a 2D material. Graphene, a single layer of carbon atoms arranged in a honeycomb lattice, offers exceptional electrical, mechanical, and thermal properties due to its large surface area and unique structure.^[^ ^163^ ^]^ The integration of graphene with NiCo_2_S_4_, a ternary metal sulfide, forms a composite material that synergistically combines the advantages of both components.

### 6.1 0D NiCo_2_S_4_ Graphene Composite Nanostructures

**Figure 2:**
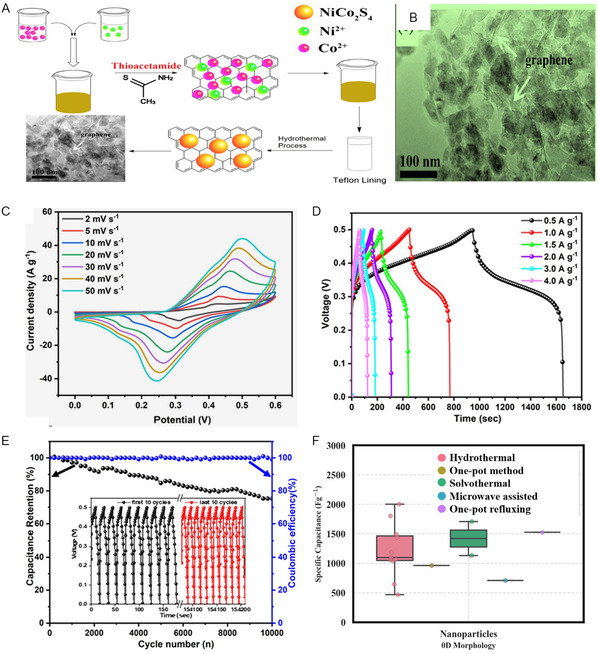
Mechanism of formation of 0D nanostructures for NiCo_2_S_4_/graphene composite. A) Schematic illustration of the fabrication processes of 1D NiCo_2_S_4_/RGO composite via one pot in situ method. B) High magnification TEM image of the NiCo_2_S_4_@GR nanocomposites, (A,B) Reproduced with permission.^[^ ^166^ ^]^ C) CV curves of NCS/graphene at different scan rates (2–50 mV s^−1^). D) GCD curves of NCS/graphene at various current densities. E) The cyclic stability and coulombic efficiency of NCS/graphene versus cycle number, (C–E) Reproduced with permission.^[^ ^167^ ^]^ F) Box plot of specific capacitance versus 0D morphology.

Decreasing the particle size of NiCo_2_S_4_ to the nanometer range offers several advantages for its performance in energy storage devices such as supercapacitors. First, it shortens the migration path length for ions and electrons within the material, improving charge transfer kinetics. Second, the increased surface‐area‐to‐volume ratio at the nanoscale provides more active sites for electrochemical reactions, boosting the specific surface area of the interfacial reaction and enabling more efficient charge storage. These enhancements result in improved reversible specific capacitance, allowing the material to store more charge per unit mass. Moreover, the nanoscale structure promotes enhanced cyclic stability, ensuring consistent performance over multiple charge and discharge cycles.^[^ ^58^, ^164^, ^165^ ^]^ The NiCo_2_S_4_/RGO composite was synthesized using a simple one‐pot in situ method, as depicted in **Figure** **2**A. NiCo_2_S_4_ nanoparticles, ranging from 20 to 50 nm in length, are intricately attached to graphene sheets, creating a conductive framework that facilitates swift and effective charge transfer. The NiCo_2_S_4_/RGO composite exhibits a specific capacitance of 1040.6 F g^−1^ at 0.2 A g^−1^, surpassing the 561.1 F g^−1^ specific capacitance achieved by pure NiCo_2_S_4_ at the same current density. The enhanced electrochemical performance can be primarily attributed to the synergistic effect of graphene and NiCo_2_S_4_ nanoparticles. The dense anchoring of numerous NiCo_2_S_4_ nanoparticles onto the highly conductive graphene sheets significantly enhances the overall electronic conductivity, enabling rapid and efficient charge transport while leveraging the pseudocapacitance of NiCo_2_S_4_ and the double‐layer capacitance of graphene. Figure 2B shows the SEM of the NiCo_2_S_4_/RGO composite.^[^ ^166^ ^]^ The microwave‐assisted method was used to synthesize NiCo_2_S_4_/graphene nanoparticles. The NiCo_2_S_4_ particles of varying sizes, stemming from diverse levels of aggregation, adhere to the graphene sheet surface, forming a structure with small spherical particles averaging 50–100 nm. As prepared hybrid electrode was tested in 3 m KOH using three three‐electrode systems. The CV study of NiCo_2_S_4_/graphene electrodes at different scan rates from 2 to 50 mV s^−1^ is shown in Figure 2C. The formation of a pair of redox peaks confirms that the energy storage mechanism primarily relies on redox reactions. The redox peaks observed in the CV curves are typically attributed to the reversible redox reactions involving Ni^2+^/Ni^3+^, Co^2+^/Co^3+^, and Co^3+^/Co^4+^. As the scan rates increased, the response currents and integral areas of the CV curves also increased. This was accompanied by slight positive shifts in the anodic peaks and negative shifts in the cathodic peaks, attributed to polarization caused by IR. The data plot of log (*i*) against log (*υ*) yields b values of 0.643 for anodic peaks and 0.684 for cathodic peaks, indicating a combination of capacitive and diffusion‐controlled behavior within the NiCo_2_S_4_/graphene composite. The Figure 2D GCD curves, obtained crosswise current densities ranging from 0.5 to 4 A g^−1^, display a nonlinear charging/discharging pattern, characterized by distinct voltage plateaus, confirming the pseudocapacitive behavior. These observations are consistent with the outcomes derived from CV analysis. Nyquist plot analysis indicated *R*_s_ value of 2.54 Ω, suggesting exceptional specific capacitance and conductivity. The small *R*_ct_ value (0.20 Ω) reflects rapid charge transfer capability, enhancing electrode performance. The plot demonstrates a linear slope in the low‐frequency region, suggesting effective ion mass transfer occurring at the interface between the electrode and electrolyte. The enduring cyclic stability of the NiCo_2_S_4_/graphene electrode was evaluated through a continuous sequence of 10,000 GCD cycles shown in Figure 2E. The decrease in capacitance retention observed during the cyclic test could be attributed to the depletion of active sites within the electrode material due to repetitive redox reactions and structural deterioration of the electrode material. Despite this, after 10,000 cycles, the capacitance retention remains at 75% of the original specific capacitance, indicating phenomenal cyclic durability. Moreover, the coulombic efficiency maintains a steady level of around 100% throughout the entire cycling process.^[^ ^167^ ^]^ Nickel‐cobalt sulfide particles embedded in graphene layers, denoted as Ni*‐*Co‐S@G were successfully prepared through a one‐step annealing process involving metallocene/MOF hybrids. This method facilitated simultaneous carbonization and sulfidation, resulting in the creation of highly conductive graphene layers with a large loading of super‐capacitive Ni*‐*Co‐S. The resulting Ni*‐*Co‐S@G composites demonstrated exceptional electrochemical performance, boasting a specific capacitance of 1463 F g^−1^ at 1 A g^−1^. Furthermore, a flexible solid‐state ASC, constructed using Ni*‐*Co‐S@G and active carbon, showed a high energy density of 51 Wh kg^−1^ at a power density of 650.3 W kg^−1^. Notably, these ASCs maintained robust flexibility and excellent performance even when bent at various angles, indicating their potential for high‐performance electrochemical capacitors. Besides, the material exhibited impressive cyclic stability, retaining 87.4% of its initial capacity following 1000 test cycles at 17 A g^−1^.^[^ ^168^ ^]^ NiCo_2_S_4_ is a material showing promise for supercapacitor applications due to its high conductivity and redox activity. However, its cyclic stability has been a concern because of structural degradation caused by redox reactions. To address this, researchers have developed a core/shell structure by encasing NiCo_2_S_4_ with an ultrathin GO layer, resulting in NiCo_2_S_4_@GO. A prepared NiCo_2_S_4_/graphene composite delivered a specific capacitance of 1100 F g^−1^ at 1 A g^−1^. When used as a positive electrode in an HSC alongside porous carbon as the negative electrode, the NiCo_2_S_4_@GO composite has shown a high energy density of 26.9 Wh kg^−1^ at a power density of 658 W kg^−1^ within the voltage range of 0 to 3.5 V. Even after 10,000 CD cycles, the device retains 78% of its initial capacitance, showing its long‐term stability. The GO coating on the NiCo_2_S_4_ surface endows the electrode with battery‐type faradaic redox properties, leading to a high specific capacitance of 1100 F g^−1^ at 1 A g^−1^ and good rate capability, with 89.1% retention of capacitance at 10 A g^−1^, as well as long cyclic stability, with 90.2% retention after 5000 cycles. Consequently, the use of NiCo_2_S_4_@GO composites as positive electrodes in ASCs shows excellent promise for supercapacitor applications, offering improved rate capacity and cyclic stability.^[^ ^169^ ^]^ Xu et al.^[^ ^78^ ^]^ developed flexible graphene supercapacitor electrodes using a biomass‐derived carbon dots/graphene composite film (CDGF) and hydrogel (CDGH), with and without an electrodeposited NiCo_2_S_4_ layer. It is revealed that the dispersed carbon dots with abundant heteroatoms effectively prevented the aggregation of graphene sheets and increased the number of active sites. The combination of carbon dots modification and NiCo_2_S_4_ electrodeposition significantly enhanced the electrochemical performance of the prepared ternary composite electrodes. Notably, the CDGF‐NiCo_2_S_4_ electrode exhibited a specific capacitance of up to 1348 F g^−1^ at a current density of 0.5 A g^−1^. Furthermore, the resultant CDGF‐NiCo_2_S_4_ flexible symmetric supercapacitor, utilizing 1M H_2_SO_4_ as an aqueous electrolyte, demonstrated a large specific capacitance (313 F g^−1^ at 0.5 A g^−1^) and a high energy density of 85.1 Wh kg^−1^ at a power density of 353 W kg^−1^. Conversely, the CDGH‐NiCo_2_S_4_ flexible solid‐state supercapacitor, using polyvinyl alcohol (PVA)/H_2_SO_4_ as a gel electrolyte, exhibited improved cyclic stability (83.1% capacitance retention after 10,000 charging/discharging cycles) and better mechanical flexibility (96.8% capacitance retention after 1000 bending cycles). Cai et al.^[^ ^61^ ^]^ synthesized NiCo_2_S_4_/RGO composite via the one‐pot reflux method. To evaluate the electrochemical properties of the NiCo_2_S_4_/RGO hybrids, GCD measurements were carried out within a voltage range of 0–0.5 V. The NiCo_2_S_4_/RGO‐3 electrode showed a specific capacitance of 1484 F g^−1^ at a current density of 2 A g^−1^. The NiCo_2_S_4_/RGO electrodes exhibit considerably higher specific capacitances than NiCo_2_S_4_ and RGO electrodes. This improved performance is attributed to the synergistic effect between NiCo_2_S_4_ and RGO. The NiCo_2_S_4_/RGO‐3 hybrids exhibit an impressive maximum specific capacitance of 1526 F g^−1^ at 1 A g^−1^ and showed excellent rate capability with a retention of 1109 F g^−1^ even at 20 A g^−1^. Moreover, after undergoing 2000 charge‐discharge cycles at 10 A g^−1^, the hybrid retains 83% of its initial capacitance, indicating robust cyclic stability. The composite of NiCo_2_S_4_ and graphene involves NiCo_2_S_4_ particles encased within an extremely thin graphene layer, creating a core/shell arrangement referred to as NiCo_2_S_4_@G. TEM analysis indicates that the graphene shell consists of merely 3–5 layers, while the NiCo_2_S_4_ particle core maintains a consistent size of ≈5–7 nm. In the charging phase, pseudocapacitance in the NiCo_2_S_4_ lattice is generated through both ionic diffusion and conversion reactions, with the latter contributing substantially more. Furthermore, an excess of ions leads to the formation of an electrochemical double‐layer capacitance on the graphene shell's surface. The NiCo_2_S_4_@G composite exhibits exceptional electrochemical performance, demonstrating a specific capacitance of 1432 F g^−1^ at 1 A g^−1^. Moreover, an HSC assembled using the synthesized NiCo_2_S_4_@G as the positive electrode and porous carbon as the negative electrode achieves a high energy density of 43.4 Wh kg^−1^ at a power density of 254.3 W kg^−1^ within the voltage range of 0–1.35 V. Even after 5000 charge/discharge cycles, the device retains an impressive 83.4% of its initial capacitance.^[^ ^170^ ^]^ Xiao et al.^[^ ^62^ ^]^ used a one‐step solvothermal approach to prepare NiCo_2_S_4_ nanoparticles with enriched edge sites decorating graphene frameworks, resulting in integrated hybrid architectures through an in situ chemically converted method. The Kirkendall effect‐driven anion exchange reaction, specifically the etching‐like action of S^2−^ ions, plays a pivotal role in forming the edge site‐enriched nanostructure. The integrated structures of Ni*‐*Co‐S nanoparticles and conductive graphene substrates, the resulting NiCo_2_S_4_/G hybrid electrodes exhibited a high specific capacitance of 1492 F g^−1^ at 1 A g^−1^, exceptional rate capability with 96% retention at an increased current density of 50 A g^−1^, and robust electrochemical stabilities. The synthesis of NiCo_2_S_4_/PRGO hybrid materials using a Phytic acid‐assisted method yielded a high‐performance battery‐type electrode for HSCs. Through the incorporation of PA and a two‐step hydrothermal process, NiCo_2_S_4_ has been effectively immobilized onto the surface of RGO sheets. The resulting NiCo_2_S_4_/PRGO electrode has demonstrated outstanding electrochemical performance, boasting a high specific capacitance of 1090 F g^−1^ at 2 A g^−1^ and exceptional cyclic stability. Likewise, when utilized as the positive electrode in an HSC device alongside AC as the negative electrode, the NiCo_2_S_4_/PRGO hybrid has exhibited a high energy density of 27.5 Wh kg^−1^ at a power density of 446.5 W kg^−1^, coupled with good cyclic stability (85.2% initial capacitance retention after 3000 cycles). These results indicate the potential of the novel NiCo_2_S_4_/PRGO hybrid material as a promising electrode material for high‐performance HSCs.^[^ ^63^ ^]^ A simple one‐step solvothermal method for the in‐situ formation of finely dispersed NiCo_2_S_4_ nanoparticles on graphene sheets was achieved without the incorporation of surfactants. The NiCo_2_S_4_@GR composite exhibits a significantly improved specific capacitance of 1708 F g^−1^ at 1 A g^−1^, compared to free NiCo_2_S_4_ which shows a capacitance of 950 F g^−1^. Furthermore, the performance of the NiCo_2_S_4_@GR electrode material can be optimized by carefully adjusting the amount of NiCo_2_S_4_ and GR in the nanocomposites. ASC devices based on these NiCo_2_S_4_@GR nanocomposites and aqueous electrolytes demonstrate a long‐term cyclic ability with a high‐energy density of 68.5 Wh kg^−1^ at 850 W kg^−1^ indicating their potential for use in high‐performance energy storage devices. The ultra‐dispersed NiCo_2_S_4_@GR nanocomposites have been synthesized in situ using an ethanolamine‐assisted solvothermal process, and the resulting ASC based on these nanocomposites exhibits a high cell voltage of 0–1.7 V in aqueous electrolyte. Due to their high electrochemical activity, specific capacitances, electric conductivity, and cell voltage of the electrolyte material, the ASC demonstrates a remarkably high energy density of 68.5 Wh kg^−1^ at a power density of 850 W kg^−1^ and an ultrahigh power density of 17 kW kg^−1^ at an energy density of 37.7 Wh kg^−1^ based on the total active material of the supercapacitor.^[^ ^165^ ^]^ The composite of NiCo_2_S_4_/RGO hybrid and NiCo_2_S_4_ hollow sphere were synthesized through a simple hydrothermal method. TEM study of synthesized composite revealed NiCo_2_S_4_ nanoparticles shows a diameter of about 20–30 nm in range and nanoparticles are uniformly developed and anchored on RGO sheet. When the experiment was performed in the absence of GO NiCo_2_S_4_ hollow spheres were obtained. In contrast, employing the same synthesis method in the presence of GO resulted in the formation of a hybrid structure with NiCo_2_S_4_ grown on GO sheets. CV study clearly showed a peak of composite material at a higher current density than NiCo_2_S_4_ and GO electrode, which indicates a synergistic effect between NiCo_2_S_4_ and GO. The NiCo_2_S_4_/RGO hybrid electrode demonstrated a significantly higher current density compared to the NiCo_2_S_4_ hollow spheres electrode and the GO electrode. This indicates a substantial improvement in electrochemical capacitance due to the synergistic effects between NiCo_2_S_4_ nanoparticles and GO sheets. The prepared material showed a specific capacitance of 1761.5 F g^−1^ at a scan rate of 2 mV s^−1^ for NiCo_2_S_4_/RGO composite. NiCo_2_S_4_ and GO showed a specific capacitance of 1076 and 183.5 F g^−1^ (2 mV s^−1^) respectively. The electrochemical capacitive properties of GO, NiCo_2_S_4_ hollow spheres, and NiCo_2_S_4_/RGO hybrid were studied using the GCD technique. GCD curves for the GO, NiCo_2_S_4_ hollow spheres, and NiCo_2_S_4_/RGO hybrid electrodes were compared at a current density of 0.5 A g^−1^. It was observed that the specific capacitances of the NiCo_2_S_4_/RGO hybrid electrode were significantly higher than those of the GO electrode and NiCo_2_S_4_ hollow spheres electrode.^[^ ^58^ ^]^ Zero‐dimensional materials, such as nanoparticles, and quantum dots often display significantly improved electrochemical performance compared to conventional bulk materials due to their high surface area and unique electronic properties.^[^ ^171^, ^172^ ^]^ However, several challenges hinder their practical applications. One major issue is the tendency for nanoparticles to agglomerate due to their ultrahigh surface energy, which can occur during synthesis, processing, or use in devices.^[^ ^61^, ^165^ ^]^ This agglomeration reduces the effective surface area available for electrochemical reactions, limiting the material's performance.^[^ ^170^ ^]^

Using ML, we conducted a stepwise analysis to investigate the impact of graphene on electrochemical performance. We systematically analysed the morphology and its relationship with specific capacitance to further elucidate this effect. Subsequently, we examined the influence of 0D, 1D, 2D, and 3D morphologies of NiCo_2_S_4_ grown on a 2D graphene sheet and their impact on the specific capacitance of the composite. This investigation involved a detailed analysis of how each morphology configuration affects the electrochemical properties relevant to supercapacitor performance. The relationship between specific capacitance and 0D morphology of NiCo_2_S_4_ composite with 2D graphene is shown in Figure 2F. The 0D NiCo_2_S_4_ consist of a nanoparticle synthesized via hydrothermal synthesis demonstrates mean specific capacitance 1209.03 F g^−1^. Notably, the solvothermal method yielded the highest single specific capacitance value of 1420.50 F g^−1^ for nanoparticles, while the one‐pot method produced promising results, the mean specific capacitance is 1526 F g^−1^ for nanoparticles. However, nanoparticles exhibit a wider specific capacitance range (468.51–2003 F g^−1^) suggesting that nanoparticles performance is more sensitive to synthesis conditions. The microwave‐assisted synthesis of nanoparticles resulted in the lowest observed specific capacitance (710 F g^−1^), potentially due to rapid reaction kinetics affecting crystal growth or rGO integration. In a conclusion, 0D NiCo_2_S_4_ nanoparticles are known for their high specific capacitance, yet research on their synthesis and enhancement is limited. Presently, these 0D NiCo_2_S_4_ nanoparticles are primarily produced via the hydrothermal method, which is quite time‐consuming. one‐step methods play an important role in synthesizing of NiCo_2_S_4_ nanoparticles anchored onto rGO sheets offer significant advantages over multi‐step approaches by reducing processing time and complexity. This approach facilitates the simultaneous formation of the NiCo_2_S_4_ nanoparticles and their integration with the rGO sheets, leading to a more intimate contact between the components. The resulting strong interface enhances electron transfer, thereby improving the overall performance of the composite electrode material. In addition, one‐step methods provide better control over the size and distribution of the NiCo_2_S_4_ nanoparticles on the rGO support, which is crucial for optimizing the electrochemical properties of the final composite. This streamlined synthesis strategy represents a promising avenue for the development of high‐performance NiCo_2_S_4_/RGO. GO sheets act as a template for NiCo_2_S_4_ nanoparticle growth. The large surface area and oxygen‐containing functional groups on GO provide nucleation sites for the initial formation of NiCo_2_S_4_ precursors. These precursors then grow and assemble around the GO sheet, eventually forming nanoparticles anchored on the RGO surface. The presence of GO sheets can restrict the diffusion of precursor molecules in the solution. This restricted diffusion can limit the ability of the precursors to collide and form larger aggregates, leading to the formation of smaller nanoparticles instead of other morphologies. Consequently, there is an opportunity to develop these nanoparticles on 2D graphene sheets using alternative chemical synthesis methods instead of the hydrothermal synthesis. The significant impact of both morphology and synthesis methods on the specific capacitance of 0D NiCo_2_S_4_/graphene composites structures, with hydrothermal and solvothermal techniques showing promise for achieving high specific capacitance values.

### 6.2 1D NiCo_2_S_4_ Graphene Composite Nanostructures

**Figure 3:**
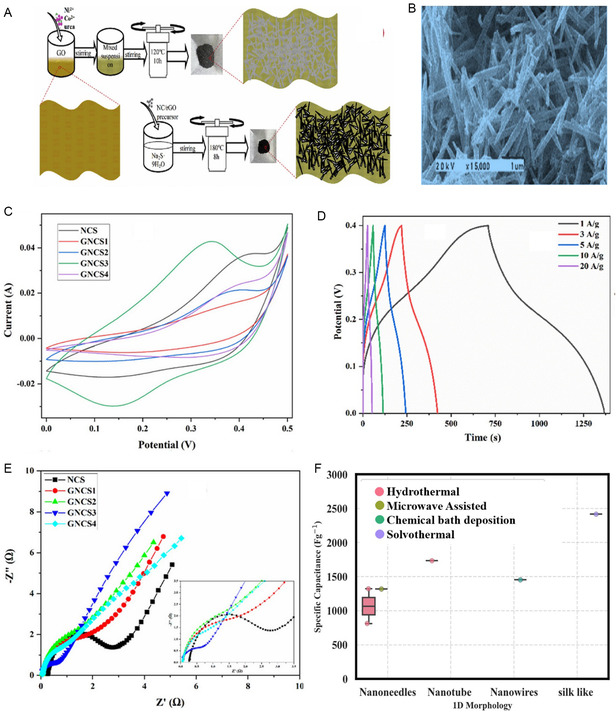
Mechanism of formation of 1D nanostructures for NiCo_2_S_4_/graphene composite. A) The synthesis diagram of composites and transformation mechanism during the synthesis process. B) SEM image of NC/rGO25S composites, (A,B) Reproduced with permission.^[^ ^173^ ^]^ C) CV curves of NCS composites at 20 mV s^−1^. D) GCD curves of GNCS3 at different current densities. E) EIS curves of NCS, GNCS1, GNCS2, GNCS3, and GNCS4, (C–E) Reproduced with permission.^[^ ^174^ ^]^ F) Box plot of specific capacitance versus 1D morphology.

NiCo_2_S_4_/rGO composites synthesized via hydrothermal process involved preparing a precursor with NiCl_2_.6H_2_O, CoCl_2_.6H_2_O, urea, and GO. The precursor, formed in a GO aqueous solution, underwent heating, washing, and freeze‐drying. Subsequently, composites were synthesized by adding the precursor to DW, Na_2_S.9H_2_O, and heating in an autoclave, yielding well‐dispersed NC/rGO precursors converted into composites with a hollow structure, labelled as NC, NC/rGO25S, NC/rGO50S, and NC/rGO100S. The schematic synthesis process of NC/rGO composites was illustrated in **Figure** **3**A accompanying the process. The formation and evolution of the composites from precursors to nickel‐cobalt sulfide/rGO composites as shown by the following chemical Equation (1) and (2)

(1) $$2Ni^{2+}+4Co^{2+}+3CH_{4}N_{2}O+12H_{2}O\to 6Ni_{1/3}Co_{2/3}{\left(CO_{3}\right)}_{0.5}OH+6NH_{4}^{+}+6H^{+}$$

(2) $$6Ni_{1/3}Co_{2/3}{\left(CO_{3}\right)}_{0.5}OH+8S^{2-}+12H^{+}\to 2NiCo_{2}S_{4}+3CO_{2}+9H_{2}O+4e^{-}$$

As graphene content increases in the composite from NCS (0.12% C) to NC/RGO 100S (23.50% C), there is a corresponding rise in carbon content, indicating graphene's contribution. The surplus carbon in NCS originates from incompletely vulcanized precursor materials. The reduction in sulfur content suggests a diminished proportion of NiCo_2_S_4_ in the composites, consistent with prior observations and discussions. The study reveals that the introduction of graphene effectively prevents the clustering of nickel‐cobalt sulfide particles. The enhanced self‐assembly of M^2+^ (Ni^2+^, Co^2+^) on RGO enriches active sites, leading to better electrochemical performance. The unique backbone structure of RGO aids in both ion transportation and electron conduction. The morphologies of NC/RGO composites are depicted in Figure 3B, with distinct features. NiCo_2_S_4_ displays an urchin‐like microsphere morphology with nanoneedles radiating from the center, while NC/RGO composites exhibit Ni–Co sulfide nanoneedles evenly dispersed on graphene, transitioning from solid nanorods to hollow nanotubes. These outcomes suggest the structural evolution and potential advantages of utilizing graphene in these composites.^[^ ^173^ ^]^ A novel hierarchical porous nanocomposite of N‐, S‐doped NiCo_2_S_4_/rGO was synthesized through the in situ growth of NiCo_2_S_4_ within a porous rGO framework via a simple hydrothermal process. The GNCS3 sample comprises NiCo_2_S_4_ nanoneedles uniformly grown on the rGO structure. These nanoneedles are evenly coated by rGO, enhancing the sample's specific surface area. The CV plot of all samples at a scan rate of 20 mV s^−1^ reveals the pseudocapacitive behavior of the materials as shown in Figure 3C with consistent shapes over samples even after the addition of rGO, indicating that the nanocomposite of rGO does not impact the charge storage mechanism of the metal sulfide material. The enclosed area within the CV curve follows the sequence: GNCS3, NCS, GNCS2, GNCS4, and GNCS1. GNCS3 demonstrates the greatest current density and exhibits improved electrochemical capacitance due to the synergistic effect of rGO and NiCo_2_S_4_ in ideal proportions. The improved electrochemical performance can be credited to the controlled amount of NiCo_2_S_4_, which hinders graphene layer aggregation and restacking. The presence of NiCo_2_S_4_ nanoneedles wedged between graphene layers, along with the formation of few‐layer graphene, enhances conductivity and surface area, thereby boosting the electrochemical performance. The GCD curve of GNCS3 illustrates capacitance values at various current densities depicted in Figure 3D. The sample GNCS3 exhibited a specific capacitance of 1640 F g^−1^ at 1 A g^−1^ at higher current densities, capacitance decreases gradually due to IR drop and limited time for ion penetration, hindering redox reactions on the electrode surface. EIS curves shown in Figure 3E display a similar shape with a semicircle and sloped line, with GNCS3 exhibiting the most vertical line, indicating faster ion transport and lower diffusion resistance ideal for supercapacitors. This can be attributed to the porous structure and high surface area of rGO layers facilitating easy ion penetration. The lower x‐intercept and smaller semicircle diameter of GNCS3 suggest low bulk resistance and rapid reaction kinetics, thanks to the high conductivity of few‐layer graphene nanosheets acting as a robust conducting framework for electron transport.^[^ ^174^ ^]^

The relationship between the specific capacitance of distinct 1D morphologies of NiCo_2_S_4_/graphene composites synthesized through different synthesis methods depicted in Figure 3F. Mostly observed nanoneedles morphology demonstrated variable performance depending on the synthesis method, with microwave‐assisted synthesis yielding a higher specific capacitance of 1320 F g^−1^ compared to the hydrothermal method (mean: 1067.17 F g^−1^, range: 813–1321.35 F g^−1^). The silk‐like structures produced via the solvothermal method exhibited the highest specific capacitance of 2418 F g^−1^, significantly outperforming other 1D morphologies. Nanotubes synthesized hydrothermally showed the second‐highest specific capacitance at 1733 F g^−1^, followed by nanowires (1454.60 F g^−1^) obtained through a two‐step chemical bath deposition. In a conclusion, the superior performance of silk‐like structures and nanotubes may be attributed to their high specific capacitance. The hydrothermal method's versatility is evident, producing three different 1D morphologies of NiCo_2_S_4_/graphene with varying specific capacitances. Notably, the microwave‐assisted method shows promise for nanoneedle synthesis, potentially due to rapid and uniform heating leads to well‐defined structures. The growth of NiCo_2_S_4_ over a graphene sheet is challenging because of thermodynamic stability and reaction conditions. The development of a specific morphology, such as 1D nanorods, nanotubes, nanowires is influenced by the thermodynamics and kinetics of the reaction. It has been noted that the development of 1D nanostructures predominantly relies on the hydrothermal method. There is a lack of extensive research on their synthesis methods to further enhance this specific capacitance. Therefore, this opens up opportunities to investigate and utilize alternative synthesis methods for synthesizing 1D nanostructures. When graphene is present, its surface energy and reaction pathways can favor the formation of advantageous morphologies, such as nanoparticles, plates, sheets or flakes, over 1D nanostructures. So, there is opportunity to developed 2D and 3D NiCo_2_S_4_/graphene composites nanostructures for achieving higher electrochemical performance.

### 6.3 2D NiCo_2_S_4_ Graphene Composite Nanostructures

**Figure 4:**
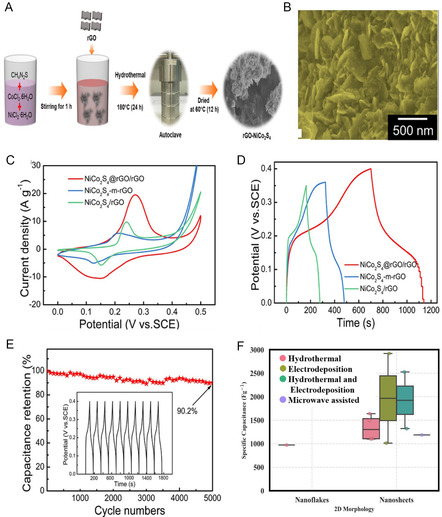
Mechanism of formation of 2D nanostructures for NiCo_2_S_4_/graphene composite A) Schematic illustration of the formation of hierarchical NiCo_2_S_4_ grown on the rGO sheets. B) FE‐SEM images of rGO‐NiCo_2_S_4_, (A,B) Reproduced with permission.^[^ ^176^ ^]^ C) CV curves of NiCo_2_S_4_@rGO/rGO at 5 mV s^−1^. D) GCD curves of NiCo_2_S_4_@rGO/rGO at 1 A g^−1^. E) cycling performance of the NiCo_2_S_4_@rGO/rGO film at 5 A g^−1^. (C‐E) Reproduced with permission.^[^ ^177^ ^]^ F) Box plot of specific capacitance versus 2D morphology.

The growth of NiCo_2_S_4_ nanosheets occurs on rGO, where rGO serves both as a substrate for growth and as a current collector for NiCo_2_S_4_ arrays. The interconnected network structure facilitates ion and electron diffusion pathways, enhancing efficient contact between ions and the active material.^[^ ^175^ ^]^ The synthesis of RGO‐NiCo_2_S_4_ was carried out via the hydrothermal method; the schematic presentation of the synthesis is depicted in **Figure** **4**A. The FE‐SEM images of NiCo_2_S_4_ illustrate the formation of a hierarchical hexagonal morphology, comprising intertwined nanoflakes. These structures with numerous open channels can enhance the contact area at the electrode/electrolyte interface, thereby improving electrolyte ion diffusivity and electron transport rate. The FE‐SEM images of RGO‐NiCo_2_S_4_ revealed that the NiCo_2_S_4_ nanoflakes were aggregated with the RGO nanosheets shown in Figure 4B. This morphology facilitates an increase in the effective contact area at the electrode/electrolyte interface, thereby enhancing the diffusivity of electrolyte ions.^[^ ^176^ ^]^ Liu et al.^[^ ^177^ ^]^ synthesized layered NiCo_2_S_4_@rGO/rGO using hydrothermal, freeze‐drying, vacuum filtration, and sulfurization/reduction techniques. NiCo_2_S_4_ nanosheets are evenly distributed on both the interior and exterior surfaces of rGO with a thickness of ≈50 nm. The CV curve analysis of NiCo_2_S_4_@rGO/rGO reveals a notably higher peak current density compared to NiCo_2_S_4_/rGO and modified (NiCo_2_S_4_‐*m*‐rGO) films, indicating its superior specific capacitance shown in Figure 4C. Moreover, a positive shift in the oxidation peak for NiCo_2_S_4_@rGO/rGO suggests a more favorable reversible redox reaction when compared to NiCo_2_S_4_‐*m*‐rGO and NiCo_2_S_4_/rGO. The GCD curves demonstrate a distinct faradaic reaction with two well‐defined voltage plateaus, pointing toward a diffusion‐controlled battery‐type faradaic process governing the energy storage mechanism shown in Figure 4D. The improved electrochemical performance primarily stems from the loose layered structure and robust interaction between NiCo_2_S_4_ and rGO nanosheets, which facilitate the diffusion of electrolyte ions/electrons and mitigate intrinsic resistance and contact resistance. The flexible electrode showcases characteristic battery‐type faradaic redox characteristics and achieves a high specific capacitance of 1100 F g^−1^ at 1 A g^−1^. The stability of the NiCo_2_S_4_@rGO/rGO film illustrated in Figure 4E consists of excellent rate capability, retaining a high specific capacitance of 943.8 F g^−1^ even after 5000 cycles at 5 A g^−1^, with a notable capacitance retention rate of 90.2%.

The specific capacitance of 2D morphologies of NiCo_2_S_4_/graphene composites, primarily nanoflakes and nanosheets, synthesized through various synthesis methods is illustrated in Figure 4F. The electrodeposition method demonstrated exceptional performance, yielding nanosheets with the highest specific capacitance of 2918 F g^−1^, with a wide specific capacitance range (1321.35–2526 F g^−1^) and a mean of 1722.90 F g^−1^, with considerable variability (standard deviation: 695.51 F g^−1^). The hydrothermal method, while versatile in producing both nanoflakes and nanosheets, showed moderate performance with nanosheets (mean: 1336.25 F g^−1^, range: 1100–1640 F g^−1^) outperforming nanoflakes (972 F g^−1^). Microwave‐assisted synthesis of nanosheets exhibits specific capacitance of 1186 F g^−1^, positioning it between hydrothermal nanoflakes and nanosheets. In a conclusion, the superior performance of electrochemically synthesized nanosheets may be attributed to their direct growth on the current collector, ensuring excellent electrical contact and facilitating efficient charge transfer. The wide performance range observed across different synthesis methods underlines the critical role of synthesis conditions in optimizing the electrochemical properties of 2D NiCo_2_S_4_/graphene composite structures. These results show the potential of electrodeposition method for achieving high specific capacitance values in 2D morphologies, while also demonstrating the versatility of hydrothermal synthesis in producing various 2D structures. Graphene sheets act as a template for NiCo_2_S_4_ growth. During synthesis, the nucleation and growth of NiCo_2_S_4_ precursors are influenced by the graphene sheet, promoting a layered growth pattern. This restricts the isotropic (equal in all directions) growth observed in free space, leading to a sheet‐like morphology. NiCo_2_S_4_ minimizes its overall surface energy when grown on graphene. By conforming to the graphene sheet, NiCo_2_S_4_ reduces the high surface energy associated with its own exposed surfaces. This favors the formation of a sheet‐like morphology that maximizes contact with graphene. The pure NiCo_2_S_4_ shows different 2D nanostructured morphologies like nanoplates, nanopetals, and nano disks via different synthesis approaches. So, there is various scope to developed remaining 2D morphologies with graphene composites of NiCo_2_S_4_ material.

### 6.4 3D NiCo_2_S_4_ Graphene Composite Nanostructures

**Figure 5:**
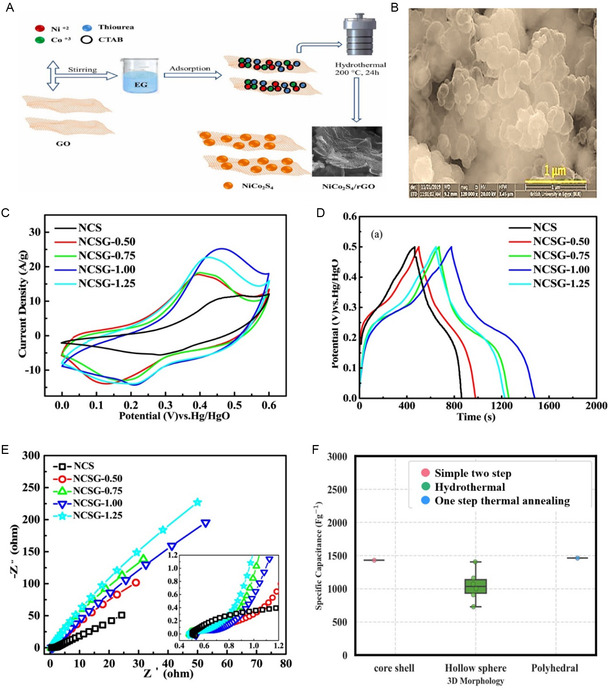
Mechanism of formation of 3D nanostructures for NiCo_2_S_4_/graphene composite A) A schematic representation shows the synthesis process of the NiCo_2_S_4_/rGO nanocomposite. B) SEM image of NiCo_2_S_4_/rGO composite, (A,B) Reproduced with permission.^[^ ^178^ ^]^ C) CV curves of NCSG composites at 10 mV s^−1^. D) GCD curves of the NCSG composites. E) EIS study NCSG composite, (C–E) Reproduced with permission.^[^ ^179^ ^]^ F) Box plot of specific capacitance versus 3D morphology.

Spinel NiCo_2_S_4_ supported on rGO was prepared using a simple one‐step hydrothermal method for energy storage purposes. This composite was compared to pristine NiCo_2_S_4_ prepared without rGO. The synthesis process of NiCo_2_S_4_/rGO refers to the schematic diagram in **Figure** **5**A. NiCo_2_S_4_ nanoparticles undergo aggregation or attachment through the oriented attachment mechanism, leading to the formation of a microsphere‐like structure with a diameter of 120 nm and a rough surface. The presence of graphene sheets plays a crucial role in preventing the aggregation of NiCo_2_S_4_, improving particle size distribution, and increasing the active surface area. The arrangement of the NiCo_2_S_4_ nanoparticles on the rGO surface significantly enhanced the supercapacitive performance of the device. The NiCo_2_S_4_ nanoparticles aggregate or attach via the oriented attachment mechanism, leading to the formation of a microsphere‐like structure with a coarse and rough surface as depicted in Figure 5B. The presence of graphene sheets prevents the aggregation of NiCo_2_S_4_, improves particle size distribution, and increases the active surface area.^[^ ^178^ ^]^ The NiCo_2_S_4_ nanospheres arranged on rGO surfaces were synthesized using a one‐step hydrothermal method. The electrochemical properties, within the potential range of 0–0.6 V, and distinct redox peaks were observed in the CV curves as shown in Figure 5C. These peaks corresponded to the redox processes involving Co^2+^/Co^3+^/Co^4+^ and Ni^2+^/Ni^3+^, as indicated by the reversible reaction. Moreover, variations in the redox potential positions of the samples were noted, influenced by the inherent phase stability of the NCS in NCSG composites. The composites exhibited lower crystallinity and inferior phase stability compared to NCS. Notably, NCSG‐1.0 demonstrated the largest integral area and peak current, suggesting superior average capacitance and Faradaic pseudocapacitor characteristics. The GCD curves of the NCSG composites displayed nonlinear behavior, indicating ongoing Faradaic reactions during the charge–discharge processes illustrated in Figure 5D. Particularly, NCSG‐1 exhibited the longest discharge time, signifying enhanced charge storage performance. Furthermore, specific capacitance values were determined to be 792 F g^−1^ for NCS and 1406 F g^−1^ for NCSG‐1 at 1 A g^−1^. The NCSG composites consistently demonstrated higher specific capacitance than NCS, attributed to the synergistic effects between the electrically conductive rGO and NCS, as previously reported. Nyquist plots of electrode samples, revealing distinct features of NCS and NCS composite, are shown in Figure 5E. All samples exhibit an unobvious semicircle at high frequencies, indicating *R*_ct_ at the electrode/electrolyte interface and double‐layer capacitance. The nonlinear low‐frequency line reflects capacitive behavior and ion diffusion resistance. NCSG composites exhibit smaller *R*_ct_ and IR compared to NCS, attributed to rGO superior electroconductivity. Moreover, NCSG composites display steeper slopes in the high‐frequency range, indicating enhanced capacitive performance and lower ion diffusion resistance, leading to higher reactivity and faster reaction kinetics.^[^ ^179^ ^]^

The literature study of 3D morphologies of NiCo_2_S_4_/graphene composites synthesized through different synthesis methods is shown in Figure 5F. The core–shell morphology, synthesized through a simple two‐step process, exhibited a specific capacitance of 1432 F g^−1^, slightly lower than the highest specific capacitance of 1463 F g^−1^ obtained from the polyhedral structure via a thermal annealing approach. Hydrothermal synthesis demonstrated versatility in producing multiple 3D structures, including nanospheres (1406 F g^−1^), porous structures (1100 F g^−1^), and hollow spheres (mean: 974.50 F g^−1^, range: 729–1161 F g^−1^). The hollow spheres showed the widest performance range among hydrothermal synthesis, suggesting sensitivity for other different synthesis conditions. The superior performance of polyhedral and core–shell structures may be attributed to their high specific capacitance, but there is a lack of extensive research on their synthesis and on ways to further improve their specific capacitance. The hydrothermal method ability to produce various 3D morphologies highlights its flexibility, although with varying degrees of specific capacitance. It has been found that 3D nanostructures are generally developed on 2D graphene sheets is often complex and requires precise control over various parameters. Notably, the thermal annealing and two‐step processes demonstrate promise for achieving high specific capacitance values in 3D structures. ML data supports the assurance that NiCo_2_S_4_/graphene composites exhibit enhanced conductivity, improved structural stability, and enhanced electrochemical performance compared to pristine NiCo_2_S_4_. In conclusion, growing NiCo_2_S_4_ nanoparticles uniformly over a large, flat graphene sheet poses a challenge. Nanoparticles tend to aggregate and form clusters, leaving behind uncovered areas on the rGO. This uneven distribution hinders the utilization of both materials and limits the overall performance of the composite. While a 3D structure offers more surface area for improved capacitance, it can also lead to a longer path for electrons to travel within the electrode. This can hinder the overall conductivity and decrease the performance of the supercapacitor. 3D structures are generally less stable than their 1D counterparts. They may be more prone to collapsing or losing their integrity during synthesis or electrochemical cycling, reducing the long‐term performance of the supercapacitor electrode.

### 6.5 NiCo_2_S_4_ Nanostructures Composite with 3D Graphene

**Figure 6:**
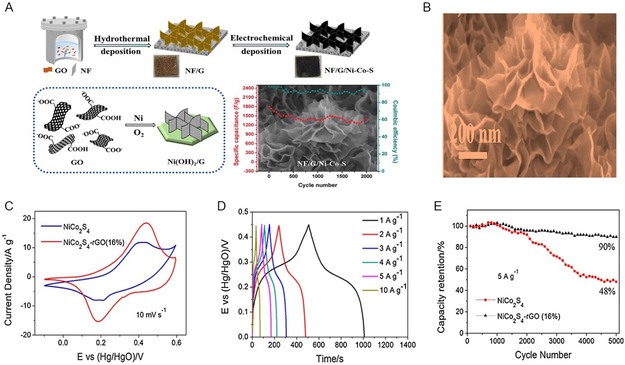
Mechanism of formation of 3D graphene nanostructures for NiCo_2_S_4_/graphene composite A) The formation process of 3D graphene. B) SEM of synthesized NF/G0.2‐KOH/Ni*‐Co*‐S, (A–B) Reproduced with permission.^[^ ^190^ ^]^ C) CV curves of the NiCo_2_S_4_ and the NiCo_2_S_4_‐rGO (16%). D) Galvanostatic charge–discharge curves of NiCo_2_S_4_‐rGO (16%) composite. E) Capacitance retention of NiCo_2_S_4_‐rGO (16%), (C–E) Reproduced with permission.^[^ ^191^ ^]^

The term “3D graphene materials” refers to engineered architectures aiming to leverage the stable and high‐performance properties of graphene through smart arrangements of graphene‐based layers or units, distinct from the graphite‐like ordering that can compromise surface area and other distinctive properties of few‐layer graphene. This distinction emphasizes the intention to preserve the unique characteristics of graphene while creating three‐dimensional structures for enhanced functionality.^[^ ^180^, ^181^, ^182^, ^183^ ^]^ The reduction of GO removes oxygen functional groups, restoring the *sp* ^2^ carbon network and promoting π‐conjugation, thereby enhancing electrical conductivity, mechanical strength, and thermal stability. Moreover, it enables the self‐assembly of RGO into intricate 3D architectures with high surface area and enhanced porosity, crucial for diverse applications including energy storage, catalysis, sensing, and tissue engineering.^[^ ^184^ ^]^ The 3D graphene (3DG) offers a higher surface area compared to 2D graphene (2DG), thereby providing more active sites for charge storage and enhancing electrochemical performance.^[^ ^185^ ^]^ Three‐dimensional (3D) graphene materials have less than 10 graphene layers arranged in a nongraphite 3D structure yet retain graphene's essential properties. These materials maintain graphene's hexagonal lattice and sp^2^ hybridization, ensuring excellent electrical conductivity, mechanical strength, and thermal conductivity. Despite their 3D configuration, they remain promising for applications like energy storage and electronics due to their graphene‐like properties.^[^ ^186^ ^]^ It is widely acknowledged that a broad distribution of pore sizes enables 3‐D graphene sheets to be adequately exposed to electrolytes, providing ample open channels for efficient electrolyte transport.^[^ ^187^, ^188^, ^189^ ^]^ The formation process of 3D graphene is illustrated in **Figure** **6**A. During the hydrothermal process, a thin layer of graphene was applied to the surface of NF through the in situ reduction and self‐assembly of graphene sheets. This was indicated by a color change on the surface from silver‐white to black. The self‐assembly of graphene occurred as GO sheets were continuously reduced, leading to the formation of cross‐linked graphene nanosheets in a 3D structure resembling graphene aerogel. Initially, GO nanosheets were dispersed in water due to their hydrophilicity and electrostatic repulsion. As the reduction progressed, the graphene became hydrophobic, facilitating the assembly process through a combination of hydrophobic and *π*–*π* interactions. The reduction degree of GO played a crucial role in determining the assembly process and morphology of the resulting graphene. Also, during the process the nickel metal oxidized to form a Ni(OH)_2_ film, with rGO covering its surface. The reductive degree of GO was found to be pivotal in the formation of 3D graphene on the NF surface, influenced significantly by the dispersion of GO. The impact of GO concentration and KOH addition on the assembly of 3D graphene nanosheets was studied. Microstructure analysis reveals that the formation of 3D graphene nanosheets on NF is closely linked to the concentration of GO and the presence of KOH. In the absence of KOH, a 3D structure of graphene platelets forms even at lower GO concentrations. Conversely, the presence of KOH facilitates the creation of a 3D porous structure consisting of graphene nanosheets on NF, with lower GO concentrations favoring the maintenance of graphene nanosheets. KOH plays a beneficial role in this assembly process by influencing dispersion during hydrothermal processing. Carboxylic ions with negative charges, resulting from ionization of carboxylic acid groups on GO sheets, are crucial in this process. The addition of KOH promotes the dispersion of GO nanosheets with negative charges, aiding in their assembly. Moreover, KOH acts as a reducing agent, creating an alkaline environment that promotes the reduction of GO. The reaction is represented by the Equation (3) and (4).

(3) $$R-COOH↔R-COO^{-}+H^{+}$$

(4) $$KOH\to K^{+}+OH^{-}\;$$

The optimal GO concentration for this assembly is found to be 0.2 mg mL^−1^, as concentrations higher or lower than this adversely affect the formation of 3D graphene nanosheets. High concentrations lead to the transformation of graphene nanosheets into platelets due to agglomeration, while lower concentrations hinder the formation of a compact 3D structure due to insufficient graphene content. The morphology and structure of the synthesized NF/G0.2‐KOH/Ni*‐Co*‐S are depicted in Figure 6B. After electrodeposition interconnected porous Ni*‐Co*‐S materials were observed to vertically envelop NF/G0.2‐KOH. The NF/G/Ni*‐*Co‐S hybrid electrode, comprising Ni*‐*Co‐S, demonstrates impressive electrochemical performance. It achieves a high specific capacitance of 2397 F g^−1^ at 5 A g^−1^, along with excellent cycle stability, retaining 77% of its capacitance after 2000 cycles at 20 A g^−1^.^[^ ^190^ ^]^ The hierarchical porous NiCo_2_S_4_/rGO composite was developed through hydrothermal treatment, combining NiCo_2_S_4_ nanosheets with 3D rGO networks. Initially, a Ni–Co precursor formed, this precursor was then uniformly dispersed in an aqueous GO solution, utilizing the *O*‐containing functional groups on GO as anchoring sites. The resulting hybrid material exhibits enhanced electron transport, a higher specific surface area, and increased active sites for redox reactions, thereby improving rate capacity and cyclic stability. During hydrothermal treatment, GO transforms into 3D rGO via self‐assembly, while NiCo_2_S_4_, derived from sulfidation of the Ni–Co precursor, uniformly attaches to the frameworks, forming a hierarchical porous structure. The typical CV curves of NiCo_2_S_4_ and NiCo_2_S_4_‐rGO (16%) reveal two distinct redox peaks at ≈0.4 and 0.2 V, as illustrated in Figure 6C. These peaks are indicative of pseudocapacitance resulting from reversible redox reactions involving Co^2+^/Co^3+^/Co^4+^ and Ni^2+^/Ni^3+^ species. Notably, the NiCo_2_S_4_‐rGO (16%) composite, featuring a hierarchical porous structure, exhibits a significantly larger CV area compared to NiCo_2_S_4_, suggesting improved energy storage capacity and faster redox kinetics. The GCD curves are depicted in Figure 6D display symmetric shapes, particularly pronounced in the NiCo_2_S_4_‐rGO hybrid electrode, indicating rapid redox processes within the electrode material. The cyclic stability analysis of NiCo_2_S_4_‐rGO (16%) is shown in Figure 6E highlights the importance of optimizing the rGO loading in composite materials to prevent a decrease in specific capacitance. Electrochemical stability assessments, involving charge/discharge tests at 5 A g^−1^ over 5000 cycles, reveal that NiCo_2_S_4_‐rGO (16%) exhibits superior cyclic stability compared to pristine NiCo_2_S_4_. The composite material demonstrates a capacitance retention of up to 90%, whereas the pristine material retains only ≈48% of its capacitance after the same number of cycles.^[^ ^191^ ^]^

In a conclusion, the 3D graphene family is rapidly expanding with numerous new structures being developed. This growth brings both challenges and opportunities. One key challenge is the need to establish comprehensive and standardized methods for property characterization, which would enable fair comparisons and significant advancements in this field. KOH acts as an etching agent. At higher KOH concentrations, it can etch away some of the carbon framework of GO, leading to the formation of defects and holes. This can promote the creation of a more open and porous 3D structure. During the hydrothermal process, KOH can also facilitate the removal of oxygen‐containing functional groups present in GO. This can lead to a more wrinkled and crumpled structure for the 3D graphene as the sheets try to minimize their surface energy. The brief study of synthesis conditions, supercapacitive properties and specific capacitance of NiCo_2_S_4_‐based graphene composite are mentioned in **Table** **1**. Summary of synthesized NiCo_2_S_4_ graphene nanostructures with different types of supercapacitor devices and their primary characteristics are mentioned in **Table** **2**.

**Table 1:** Study of synthesis conditions, supercapacitive properties, and specific capacitance of NiCo_2_S_4_‐based graphene composite.

Sr. No.	Synthesis condition	Method	Morphology	Voltage range [V]	Specific capacitance/capacity	Electrolyte	Surface area	Capacitance retention	References
1.	Ni(NO_3_)_2_·6H_2_O + Co(NO_3_)_2_·6H_2_O + HMT (molar ratio of 1:2:4.4) + 30 mL DW + 15 mL ethanol + heated at 80 °C for 6 h + 0.5 g Na_2_S·9H_2_O + 30 mL GO + heated at 160 °C for 8 h + freeze‐dried	Hydrothermal method	Nanosheets	0–0.6	1107 F g^−1^ at 1 A g^−1^	6 m KOH	38.4 m^2 ^g^−1^	90% after 5000 cycles at 5 A g^−1^	[191]
2.	As prepared pure rGO/Ni, Co‐hydroxide precursor + 30 mL DW + 0.3 g thiourea + heated at 160 °C for 1 day + vacuum filtration + freezing dried by heating at 180 °C for 30 min under N_2_ atmosphere	Hydrothermal method	Nanoparticles	0–0.6	1059 F g^−1^ at 2 A g^−1^	2 m KOH	28.57 m^2 ^g^−1^	87.3% after 3000 cycles at 20 A g^−1^	[237]
3.	0.1 g GO powder + 40 mL water + (40, 60, 80, 100 mmol) Co(NO_3_)_2_·6H_2_O + same amount of thiourea + 40 mL solution + (40, 60, 80, 100 mmol) Ni(NO_3_)_2_·6H_2_O + same amount CH_3_CSNH_2_ + both solutions mixed + heated at 180 °C for 24 h + dried at 70 °C for 12 h	Hydrothermal method	3D Hierarchical composites with sandwiched structure	0–0.6	2003 F g^−1^ at 1 A g^−1^	2 m KOH	32.1 m^2 ^g^−1^	86% after 3500 cycles	[238]
4.	20 mg GO + equal amount of water + 0.1 mm Ni(acetate)_2_ · 4H_2_O +1 mm Co(acetate)_2_ · 4 H_2_O + 20 mL ethanol + 0.6 mm Na_2_Co_3_ + 1.5 mL di‐isopropyl amine + heated at 120 °C for 16 h + redispersed in 0.25 mm Na_2_S · 9H_2_O + heated at 160 °C for 10 + dried at 60 °C + annealed at 200 °C at 1 h under N_2_ atmosphere	Hydrothermal method	Ultrathin nanosheets structure	0–0.5	1498 F g^−1^ at 1 A g^−1^	1 m KOH	111 m^2 ^g^−1^	89.1% after 100,000 cycles	[160]
5.	1 mmol Ni(NO_3_)_2_ · 6H_2_O + 2 mmol Co(NO_3_)_2_ · 6H_2_O + 4 mmol thiourea + 2 mL EN + 30 mg GO + 30 mL H_2_O + heated at 200 °C for 12 h + dried at 60 °C	Hydrothermal method	Hollow spheres are constructed by interconnected nanosheets	0–0.5	1161 F g^−1^ at 5 A g^−1^	2 m KOH	–	High cycling stability	[161]
6.	60 mL GO suspension + as prepared NiCo_2_S_4_ hollow spheres + vacuum filtering the mixed suspension on a polycarbonate membrane with 200 nm pores + under N_2_ atmosphere at 400 °C for 2 h + samples with mass ratios NiCo_2_S_4_ to RGO of (1:2 Volume)	Hydrothermal method	Flexible yolk‐shelled hollow spheres	0–0.5	1000.5 F g^−1^ at 1 A g^−1^	6 m KOH	26.62 m^2 ^g^−1^	Good cyclic stability	[239]
7.	10 mg graphite oxide + 20 mL DW + 5 mL NaOH solution (0.5 m) + refluxed at 95 °C for 1 h + pre‐prepared NiCo_2_S_4_ aqueous dispersion + vacuum dried at 45 °C	One‐step hydrothermal method	Nanocomposites	0–0.5	1437.5 F g^−1^ at 2 A g^−1^	3 m KOH	8.78 m^2 ^g^−1^	High cyclic stability	[240]
8.	1.855 g CoCl_2_ + 0.95 g NiCl_2_ + 0.72 g urea and 60 mL DW + pre‐prepared NF + heated at 120 °C for 6 h + dried at 50 °C for 4 h + 1.8 g Na_2_S + 60 mL DW + heated at 180 °C for 8h	Hydrothermal method	3D core/shell structure	0–0.55	11.6 F cm^−2^ at 100 mA cm^−2^	3 m KOH	–	93% after 5000 cycles at 10 mA cm^−2^	[241]
9.	0.76 g NiCl_2_ · 6H_2_O + 0.38 g CoCl_2_ · 6H_2_O + 6.08 g thiourea + (deaerated by purified nitrogen purging for 30 min + CV was used for deposition at a rate of 5 mV s^−1^ in the range of −1.2 to 0.2 V for 24 cycles + dried overnight at 60 °C	Electrodeposition method	Composite nanosheet structure	0–0.6	1013 F g^−1^ at 0.5 A g^−1^	2 m KOH	–	High cyclic stability	[242]
10.	As prepared Ni‐Co precursor + 15 mmol urea + 5 mmol TAA + heated at 90 °C for 5 h + 140 °C for 5 h + vacuum dried at 60 °C overnight	Two‐step hydrothermal method	Nanotube arrays	0–0.6	1733 F g^−1^ at 1 A g^−1^	6 m KOH	–	87% after 5000 cycles	[243]
11.	As prepared Ni‐Co precursor + (TAA, 0.17 g) + (30 mg) GO + 60 mL ethanol + heated at 120 °C for 6 h + dried at 60 °C + annealed at 350 °C for 2 h in N_2_ atmosphere	Solvothermal method	Nanoparticles	0–0.45	1133 F g^−1^ at 1 A g^−1^	6 m KOH	54.9 m^2 ^g^−1^	High cyclic stability	[244]
12.	30 mg GO powder + 30 mL DW + 1 mmol of NiCl_2_ · 6H_2_O + 2 mmol CoCl_2_ · 6H_2_O + 4 mmol (TSC) thiosemicarbazone + heated at 180 °C for 12 h + vacuum dried at 60 °C	Hydrothermal method	Nanoparticles	0–0.4	1804.7 F g^−1^ at 0.5 A g^−1^	2 m KOH	–	Good electrochemical stability	[58]
13.	60 mg GO + 60 mL DW + 2 mmol Ni(NO_3_)_2_ · 6H_2_O + 4 mmol Co(NO_3_)_2_ · 6H_2_O + 5 mmol HMT + 20 mL water + heated at 90 °C for 6 h + freeze‐dried for 48 h + 40 mL 0.05 m Na_2_S solution + 160 °C for 6 h + dried	Hydrothermal method	Nanosheet	0–0.5	1100 F g^−1^ at 1 A g^−1^	6 m KOH	–	Good cyclic stability	[177]
14.	30 mg GO + 30 mL EG + 0.15 mmol Ni(NO_3_)_2_ · 6H_2_O + 0.30 mmol Co(NO_3_)_2_ · 6H_2_O + 0.90 mmol CS_2_ + heated at 150 °C for 15 min + vacuum dried at 80 °C for 24 h	One‐step microwave‐assisted method	Nanoneedles	0–0.6	1320 F g^−1^ at 1.5 A g^−1^	6 m KOH	–	96% after 2000 cycles	[239]
15.	0.2 mmol nickel (II) acetylacetonate [Ni(acac)_2_]_3_ + 0.4 mmol cobalt (III) acetylacetonate (Co(acac_2_)_3_ + 1 mL oleic acid + RGO + 7.5 mL OLA + heated to 100 °C for 15 min + heated at 100 °C for optimal sulfur:cation ratios from 1:1 to 3:1 + heated at 250 °C + maintained for 20, 40, 60, and120 min resp. + calcinated under argon atmosphere at 350 °C for 1 h	One‐pot method	Nanocomposite	0–0.6	963 F g^−1^ at 1 A g^−1^	2 m KOH	–	70% after 3000 cycles	[164]
16.	60 mg rGO powder + 60 mL DW + 1 mmol (Ni(NO_3_)_2_ · 6H_2_O) + 2 mmol (Co(NO_3_)_2_ · 6H_2_O) + 4 mmol thiocarbamide + heated at 180 °C for 12 h + freeze‐dried for 24 h	One‐step hydrothermal method	Nanospheres	0–0.6	1406 F g^−1^ at 1 A g^−1^	6 m KOH	–	82.36% after 2000 cycles at 1 A g^−1^	[179]
17.	20 mg GO + 60 mL EG + molar ratio (Ni(Ac)_2_·4H_2_O:Co(Ac)_2_·4H_2_O) (1:2) + 6 mmol thiourea + refluxed at 180°C for 3 h + vacuum dried at 45 °C for 12 h	One‐pot refluxing method	Nanoparticles	0–0.6	1526 F g^−1^ at 1 A g^−1^	3 m KOH	34.1 m^2 ^g^−1^	83% after 2000 cycles at 10 A g^−1^	[61]
18.	1 mmol Ni(NO_3_)_2_ · 6H_2_O + 2 mmol Co(NO_3_)_2_ · 6H_2_O + 9 mmol thiourea + GO aqueous dispersion + 1 mL ammonia + 25 mg CNT + 30 mL DW + heated at 180 °C for 24 h + vacuum dried at 60 °C for 12 h	Hydrothermal method	Nanoparticles	−0.1 to 0.4	468.51 F g^−1^ at 2 A g^−1^	6 m KOH	–	80.5% over 5000 cycles	[175]
19.	300 mg as prepared (Ni, Co) EDTA precursors + 40 mL ethanol + 0.5 g TAA + heated at 120 °C for 6 h + EDTA‐NiCo_2_S_4_ annealed under N_2_ atmosphere at 550 °C for 2 h	Simple two‐step process	Core/shell structure	0–0.45	1432 F g^−1^ at 1 A g^−1^	2 m KOH	–	–	[170]
20.	20 mg GO + 20 mL DW + 1 mmol Co(NO_3_)_2_·6H_2_O + 0.5 mmol Ni(NO_3_)_2_·6H_2_O + 6 mmol thiourea + 20 mL EA + heated at 200 °C for 14 h + dried at 60 °C for 12 h	One‐step solvothermal approach	Nanocomposites	−0.1 to 0.45	1708 F g^−1^ at 1 A g^−1^	6 m KOH	–	96.7% over 5000 Cycles	[165]
21.	As‐synthesized Ni‐Co precursor/G + 40 mL water:TAA ratio (1:2) + heated at 180 °C for 12 h	Hydrothermal method	Nanoparticles	0–0.6	1492 F g^−1^ at 1 A g^−1^	6 m KOH	25 m^2 ^g^−1^	90% after 8000 cycles	[62]
22.	3 mmol nickel acetate + 6 mmol cobaltous acetate + 18 mmol TAA + 1.5 mg mL^−1^ graphene suspension (100 mL) + heated at 200 °C for 6 h + dried at 60 °C overnight	Hydrothermal method	Microstructure	0–0.5	1040.6 F g^−1^ at 0.2 A g^−1^	6 m KOH	–	89.3% after 2000 cycles	[166]
23.	25 mg Ni(CH_3_CO_2_)_2_·4H_2_O + 25 mg CoCO_3_ + 10 mg sulfur powder + 60 mg graphene + heated by a microwave oven for 60 s + decomposition + irradiation process	Ultrafast microwave solid‐state Synthesis	Nanoparticles	0–0.6	710 F g^−1^ at 0.5 A g^−1^	3 m KOH	–	75% after 10,000 cycles	[167]
24.	75 mg GO + 25 mL ethanol + 10 mL DW + 46 mg Ni(Ac)_2_ + 92 mg Co(Ac)_2_ + 167 mg thiourea + 25 mL EG + heated at 200 °C for 8 h + freeze‐vacuum dried for 30 h	One‐step hydrothermal reaction	Particles underwent severe aggregation	−0.2 to 0.5	645 F g^−1^ at 20 A g^−1^	6 m KOH	–	Good cyclic stability	[60]
25.	As prepared Ni‐Co precursor/RGO + 0.07 m Na_2_S solution (40 mL) + heated at 160 °C for 8 h + dried at 60 °C for overnight	Two‐step hydrothermal method	Hollow spherical shells assembled by nanoparticles	0–0.45	729 F g^−1^ at 2 A g^−1^	6 m KOH	89.5 m^2 ^g^−1^	68.8% after 3000 cycles	[63]
26.	200 mg as prepared precursor (NC/rGO, NC) + 100 mL DW + 7 mmol (1.7156 g) Na_2_S · 9H_2_O + heated at 180 °C for 8 h	Hydrothermal synthesis method	Hollow structure	0–0.5	910 F g^−1^ at 2 A g^−1^	6 m KOH	50.60 m^2 ^g^−1^	Good cyclic stability	[173]
27.	15 mL of aqueous solution containing 0.5 mmol Ni(NO_3_)_2_·6H_2_O + 1 mmol Co(NO_3_)_2_·6H_2_O + 3 mmol urea + 22 mL of graphene dispersion (1.6 mg mL^−1^) + heated at 80 °C for 2 h + 2.25 mmol thiourea + 1 mmol NH_4_F + heated at 180 °C for 2 h + vacuum dried at 60 °C for 12 h	One‐step hydrothermal method	Nanoparticles decorated on the surface of graphene nanosheets	0–0.6	1063 F g^−1^ at 2 A g^−1^	1 m KOH	55 m^2 ^g^−1^	82% after 10,000 cycles	[56]
28.	As pre‐treated pure graphene film (GF) + 5 mm Ni(NO_3_)_2_·6H_2_O + 10 mm of Co(NO_3_)_2_·6H_2_O + 0.75 m CS(NH_2_)_2_ + maintained at a current density of 5 A g^−1^ for 800 s	Waterbath heating and hydrothermal methods	Nanoparticles on graphene sheet	0–0.5	1186 F g^−1^ at 0.5 A g^−1^	1 m H_2_SO_4_	–	Good cyclic stability	[78]
29.	0.2 m NiCl_2_ · 6H_2_O + 0.4 m CoCl_2_ · 6H_2_O + 1.2 m CH_4_N_2_S + 1.6 m 2‐MI + 30 mL DDW + 30 mg graphene oxide + heated at 180 °C for 24 h + dried at 60 °C for 12 h in air	One‐pot hydrothermal method	Nanoflakes aggregated with the rGO nanosheets	−0.1 to 0.45	972 F g^−1^ at 1 A g^−1^	6 m KOH	–	70.58% after 2000 cycles at 20 A g^−1^	[176]
30.	45 mg GO + 15 mL DW + 0.20 mmol Ni(NO_3_)_2_·6H_2_O + 0.40 mmol Co(NO_3_)_2_·6H_2_O + 1.2 mmol thiourea + heated at 180 °C for 5 h + freeze‐dried	One‐step hydrothermal method	Needle‐like attached to graphene sheets	−0.1 to 0.5	813 F g^−1^ at 1.5 A g^−1^	3 m KOH	62.2 m^2 ^g^−1^	–	[245]
31.	As prepared NiCo_2_(CO_3_)_1.5_(OH)_3_ NWs + 0.625 g Na_2_S·9H_2_O (2.6 mM) + 50 mL DW + heated at 160 °C for 6 h + dried at 65 °C for 10 h	Two step chemical bath deposition	Nanowire arrays	0–0.5	1454.6 F g^−1^ at 1.3 A g^−1^	2 m KOH	–	96% after 3000 cycles	[246]
32.	500 mg of sulfur powder + 100 mg nickelocene/MOFs powder + heated at 600 °C for 2 h under an Argon atmosphere	One‐step thermal annealing approach	Polyhedral morphology	0–0.3	1463 F g^−1^ at 1 A g^−1^	6 m KOH	42.5 m^2 ^g^−1^	87.4% after 1000 cycles	[168]
33.	5 mmol Ni(CH_3_COO)_2_·4H_2_O + 10 mmol Co(CH_3_COO)_2_·4H_2_O + 30 mL EG + 15 mg GO + 60 °C for 2 h + 20 mmol thiourea + heated at 200 °C for 12 + dried at 60 °C for 24 h	Facile solvothermal method	Silk‐like morphology	0–0.7	2418 F g^−1^ at 1 A g^−1^	6 m KOH	–	Good cyclic stability	[247]
34.	As prepared rG/NC‐LDH film + heated at 160 °C for 6 h + 1.8 g thiourea + 60 mL DW + 24 mL ethanol + 160 °C for 6 h	Hydrothermal method	Nanoneedle‐like arrays structure	−0.15 to 0.55	–	6 m KOH	–	Good cyclic stability	[248]
35.	20 mL GO solution (0.2 mg mL^−1^) + deposition potential is −1.2 V + reaction time is 5 min	–	Nanosheet array	−0.2 to 0.5	–	6 m KOH	–	72.3% after 5000 cycles	[249]
36.	GO + 50 mL ethanol + as prepared NiCo_2_S_4_ + heated at 140 °C for 18 h + dried at 100 °C	Hydrothermal method	Agglomeration and pores‐like structure	0–0.5	1100 F g^−1^ at 1 A g^−1^	6 m KOH	49.2 m^2 ^g^−1^	90.2% after 5000 cycles	[169]
37.	5 mmol L^−1^ CoCl_2_·6H_2_O + 0.075 mol L^−1^ thiourea + at different concentrations of 5, 7.5, and 10 mmol L^−1^ NiCl_2_·6H_2_O + ammonic solution + CV applied at a scan rate of 5 mV s^−1^ for 15 cycles within a potential range from −1.2 to 0.2 V + drying in air for 6 h + vacuum dried at 80 °C for 6 h	One‐step electrochemical method	Nanosheets structure	−0.2 to 0.6	2918 F g^−1^ at 1 A g^−1^	1 m KOH	–	Good cyclic stability	[250]
38.	As prepared rGO/NiCo‐carbonate hydroxide (GNC) precursor + 40 mL DW + 600 mg Na_2_S + heated at 180 °C for 8 h + dried at 70 °C	Hydrothermal process	Sheet‐like morphology	0–0.5	1640 F g^−1^ at 1 A g^−1^	–	254.18 m^2 ^g^−1^	Good cyclic stability	[174]
39.	0.12 g cetyltrimethylammonium bromide + 0.01 g GO + 60 mL EG + NiCl_2_·6H_2_O + CoCl_2_.6H_2_O + thiourea (molar ratio of 1:2:4) + heated at 200 °C for 24 h + dried at 80 °C for 12 h	Hydrothermal method	Hollow microsphere‐like structure	−0.1 to 0.6	1072 F g^−1^ at 1 A g^−1^	6 m KOH	14.21 m^2 ^g^−1^	Good cyclic stability	[178]
40.	5 mm CoCl_2_·6H_2_O + 7.5 mm NiCl_2_·6H_2_O + 0.75 M (CS(NH_2_)_2_ + NF/G film + at room temperature by using CV at a scan rate of 5 mV s^−1^ for 15 cycles + voltage range of −1.2 V to 0.2 + vacuum dried at 80 °C for 24 h	Hydrothermal and electrodeposition method	Nanosheet array's structure	−0.2 to 0.6	2526 F g^−1^ at 2 A g^−1^	6 m KOH	–	77% after 2000 cycles	[190]
41.	0.12 g graphene + 50 mL DW + 4 mmol Ni(NO_3_)_2_·6H_2_O + 8 mmol Co(NO_3_)_2_·6H_2_O + 17.2 mmol HMT + 20 mL ethanol + microwave radiation at 600 W for 20 min + 16 mmol TAA + 70 mL DW + heated at 250 °C for 2 h under argon atmosphere	Microwave‐induced synthesis	Honeycomb‐like nanosheet structure	0–0.6	1186 F g^−1^ at 1 A g^−1^	3 m KOH	50.5 m^2 ^g^−1^	Good cyclic stability	[206]

**Table 2:** Summary of synthesized NiCo_2_S_4_ graphene nanostructures with different types of supercapacitor devices and their primary characteristics.

Sr. No.	Device	Voltagerange [V]	Electrolyte	Energydensity[Wh kg^−1^]	Powerdensity[W kg^−1^]	Specific capacitance/capacity	Capacitance retentionafter cycle	Devicetype	References
1.	NiCo_2_S_4_‐rGO//NCCF‐rGO	0–1.6	6 m KOH	36	1600	–	95% after 8000 cycles	ASC	[191]
2.	rGO/NiCo_2_S_4_//rGO/MnO	0–1.6	2 m KOH	38.8	0.4 kW kg^−1^	100 F g^−1^ at 0.5 A g^−1^	75% after 5000 cycles	ASC	[237]
3.	NiCo_2_S_4_@RGO//AC	0–1.8	2 m KOH	13.5	2700 kW kg^−1^	63 F g^−1^ at 0.1 A g^−1^	88.9% after 2200 cycles	ASC	[238]
4.	NiCo_2_S_4_@rGO//G‐SWCNHs	0–1.6	1 m KOH	60.9	1.4 kW kg^−1^	171.3 F g^−1^ at 1.8 A g^−1^	89.1% over 10,000 cycles	ASC	[160]
5.	NiCo_2_S_4_/2RGO//AC	0–1.6	6 m KOH	15.4	2227.3	43.4 F g^−1^ at 1 A g^−1^	80.5% after 5000 cycles	ASC	[162]
6.	NCS/CNS//RGO	0–1.6	3 m KOH	23.9	2460.6	15.6 F cm^−2^	93% after 5000 cycles	ASC	[241]
7.	NiCo_2_S_4_/RGO	0–1.6	2 m KOH	24.4	750	78 F g^−1^ at 1 A g^−1^	Good cyclic stability	ASC	[58]
8.	NiCo_2_S_4_@rGO/rGO	0–1.8	6 m KOH	30.0	857.6	70.6 F g^−1^ at 1 A g^−1^	78.5% after 2500 cycles	ASC	[177]
9.	RGO/NiCo_2_S_4_//RGO	0–0.8	6 m KOH	46.7	1200.8	146 F g^−1^ at 1 A g^−1^	88.9% after 1500 cycles	ASC	[239]
10.	NiCo_2_S_4_/RGO//AC	0–1.6	2 m KOH	23	7418	62 F g^−1^ at 2 A g^−1^	90% after 1800 cycles	ASC	[164]
11.	NiCo_2_S_4_@G//PC	0–1.35	2 m KOH	43.4	254.3	170.7 F g^−1^ at 1 A g^−1^	83.4% after 5000 cycles	HSC	[170]
12.	NiCo_2_S_4_@GR//AC	0–1.7	6 m KOH	68.5	850.0	170.6 F g^−1^ at 1 A g^−1^	95.8% after 5000 cycles	ASC	[165]
13.	Ni*‐Co*‐S/G//PCMS	0–1.6	2 m KOH	28.4	22.1 kW kg^−1^	122 F g^−1^ at A g^−1^	85% after 10,000 cycles	ASC	[62]
14.	NiCo_2_S_4_/graphene	0–1.4	6 m KOH	2.9	872	–	–	ASC	[166]
16.	NCS/graphene//AC	0–1.6	3 m KOH	30.29	400	85 F g^−1^ at 0.5 A g^−1^	112% after 10,000 cycles	ASC	[167]
17.	NiCo_2_S_4_/PRGO//AC	0–1.6	6 m KOH	27.5	446.5	77.4 F g^−1^ at 2 A g^−1^	85.2% after 3000 cycles	HSC	[63]
18.	NC/rGO25S	0–1.6	6 m KOH	–	–	199.3 F g^−1^ at 2 A g^−1^	90.4% after 10,000 cycles	ASC	[173]
19.	NiCo_2_S_4_/GNS//NG	0–1.6	1 m KOH	54.6	350.8	175 F g^−1^ at 0.5 A g^−1^	80% after 8000 cycles	ASC	[56]
20.	CDGF‐NiCo_2_S_4_//CDGF‐NiCo_2_S_4_	0–1.4	1 m H_2_SO_4_	85.1	353	313 F g^−1^ at 0.5 A g^−1^	83.1% after 10,000 cycles	flexible SSC	[78]
21.	rGO‐NiCo_2_S_4_//AC	0–1.4	6 m KOH	56.62	701.37	208 F g^−1^ at 1.4 A g^−1^	94.1% after 2000 cycles	ASC	[176]
22.	RGO/NiCo_2_S_4_//RGO	0–1.5	3 m KOH	40.3	375.0	45.3 F g^−1^ at 1 A g^−1^	84.3% after 2000 cycles	ASC	[245]
23.	Ni‐Co‐S@G//AC	0–1.3 L	6 m KOH	51.0	650.3	217.8 F g^−1^ at 1 A g^−1^	93.3% after 1000 cycles	ASC	[168]
24.	NiCo_2_S_4_@rGO//N‐rGO	0–1.6	6 m KOH	34.1	411	–	86.4% after 5000 cycles	ASC	[247]
25.	rG/NCS/NCO//rGO	0–1.5	6 m KOH	32	412	104 F g^−1^ at 3 A g^−1^	65% after 1000 cycles	ASC	[248]
26.	r‐GDYO/NiCo_2_S_4_//AC	0–1.5	6 m KOH	0.41 mWh cm^−2^	7.24 mW cm^−2^	1.42 F cm^−2^ at 30 mA cm^−2^	92.1% after 5000 cycles	–	[249]
27.	NiCo_2_S_4_@GO//AC	0–1.8	2 m KOH gel	26.9	658	83.6 F g^−1^ at 1 A g^−1^	78% after 10,000 cycles	ASC	[169]
28.	GNCS‐3//AC	–	–	27	600	135 F g^−1^ at 1 A g^−1^	92.5% after 5000 cycles	ASC	[174]
29.	NiCo_2_S_4_/RGO//AC	0–1.8	6 m KOH	41.52	1067	103.5 F g^−1^ at 1 A g^−1^	82% after 3000 cycles	HSC	[178]
30.	NCS/G‐H//AC	0–1.6	3 m KOH	46.4	400	130.5 F g^−1^ at 0.5 A g^−1^	89.2% after 10,000 cycles	HSC	[206]
31.	Ni‐Co–S/GF//PPy/GF	0–1.65	1 m KOH	37.7	16 100	209.82 F g^−1^ at 1 A g^−1^	54.02% after 5000 cycles	ASC	[251]

The unique properties of both two 2D and 3D graphene‐based materials contribute to improved electrochemical performance in various applications. The different graphene morphologies play a critical role in directly influencing both the scalability of NiCo_2_S_4_/Graphene composites and their suitability for industrial applications. The choice between predominantly 2D graphene nanosheets, 3D graphene architectures (like foams or aerogels), or even crumpled graphene structures significantly impacts the ease and cost‐effectiveness of composite synthesis. For instance, while 2D graphene offers high surface area for NiCo_2_S_4_ deposition, its tendency to restack can hinder scalability due to challenges in achieving uniform dispersion and consistent composite formation in large volumes. Conversely, 3D graphene frameworks, although potentially more complex to synthesize initially, can provide a robust and interconnected scaffold that facilitates uniform NiCo_2_S_4_ growth and prevents graphene aggregation, potentially leading to more scalable and reproducible production processes.^[^ ^192^ ^]^ GO exists in different morphologies, including crumpled graphene, exfoliated graphene, and hybrid graphene. Crumpled graphene flakes or rGO spheres exhibit high surface area and tunable pore structures, making them suitable for applications requiring enhanced mechanical strength, such as metal matrix composites. These morphologies are particularly useful in forming interconnected networks that improve strength and conductivity.^[^ ^193^, ^194^ ^]^ Exfoliated graphene layers dispersed homogeneously in polymer matrices maximize the interfacial contact area, leading to superior mechanical and thermal properties. This morphology is preferred for polymer nanocomposites used in aerospace and automotive industries. Exfoliated graphene supercapacitors have shown tremendous potential for energy storage applications due to their high specific capacitance, excellent rate capability, and long cycling stability. Various synthesis methods, including electrochemical exfoliation, thermal exfoliation, and electrophoretic deposition, have been developed to produce high‐quality graphene nanosheets. Hybrid systems involving graphene wrapped around nanoparticles or integrated with polymers allow for tailored properties like high surface area and improved electroactivity. Such morphologies are scalable using techniques like colloidal coagulation or catalytic synthesis.^[^ ^194^, ^195^, ^196^ ^]^ Furthermore, the morphology dictates the final composite's properties, such as electrical conductivity, mechanical stability, and electrolyte accessibility. The composite with well‐dispersed NiCo_2_S_4_ on a conductive and porous 3D graphene network might exhibit superior electrochemical performance and durability, making it more attractive for large‐scale energy storage solutions compared to a composite with poorly dispersed NiCo_2_S_4_ on restacked 2D graphene, which might suffer from limited ion diffusion and lower cycle life. The synthesis of 3D graphene is more complex as compared to 2D graphene. Therefore, tailoring the graphene morphology is not only essential for optimizing the composite's performance but also for ensuring its cost‐effective and consistent production at an industrial scale, ultimately determining its viability for real‐world applications. One of the primary challenges in synthesizing 3D graphene is scaling up the production process while maintaining material quality. Current methods often struggle to produce large quantities of 3D graphene without compromising its structural integrity or electrical properties. For instance, while laboratory‐scale synthesis can yield high‐quality 3D graphene, transitioning to industrial‐scale production requires addressing issues such as uniformity, consistency, and cost‐effectiveness.^[^ ^197^, ^198^ ^]^

## 7 Electronic Structural Investigation of NiCo_2_S_4_/Graphene Composites

The advancement of energy storage technologies, particularly supercapacitors, necessitates a deep understanding of the materials used in their construction. DFT is a pivotal tool for predicting and analyzing material properties. Classical DFT evaluates the structural and porous characteristics of carbon‐based materials, while electronic DFT elucidates the electronic properties crucial for energy storage performance. Electronic DFT can estimate quantum capacitance by analyzing the density of states (DOS) of electrode materials, and classical DFT provides insights into the electrode/electrolyte interface by calculating the electrical double layer capacitance.^[^ ^199^, ^200^, ^201^ ^]^ DFT simplifies computational complexity by focusing on charge densities rather than many‐body wave functions, with the total energy of a system determined solely by its electron density. This streamlines calculations, facilitating the development of advanced supercapacitors. The LDA,^[^ ^202^, ^203^ ^]^ and GGA are key methods within DFT, with the latter improving accuracy by considering the electron density gradient. Enhancing quantum capacitance through surface defects, doping, or functional groups is often explored using DFT simulations, providing insights into structural, electronic, and charge transfer mechanisms. DFT calculations, including spin‐polarized configurations and first‐principle theoretical calculations, aid in understanding and optimizing the properties of materials like NiCo_2_S_4_, further advancing supercapacitor technology.^[^ ^204^, ^205^ ^]^

**Figure 7:**
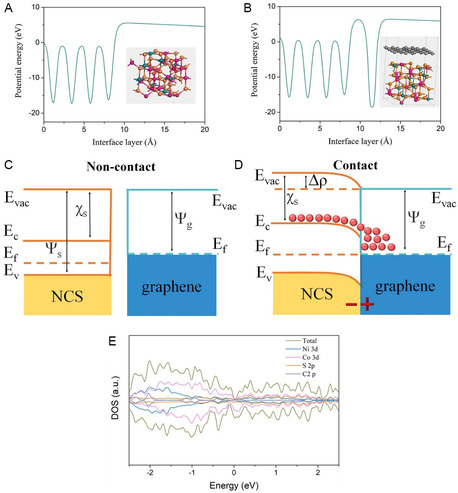
DFT study of NiCo_2_S_4_ nanostructures and its graphene composites; A) potential energy of (A) NCS surface, B) NCS/graphene surface (the inset is the atom model of NCS or NCS/graphene). Energy band schematic illustration of NCS–graphene interface, C) noncontact, D) contact. Where *E*_v_ is the maximum energy of the valence band, *E*_vac_ is the vacuum energy, *E*_c_, is the lowest energy of the conduction band, *E*_f_ is the Fermi level, *χ*_s_ is the electron affinity, Ψ_s_ is the work function, and Δ*ρ* is the work function difference. E) LDOS of NCS‐graphene, (A–E) Reproduced with permission.^[^ ^206^ ^]^

The stability of the NiCo_2_S_4_/graphene composite under operational conditions can be evaluated using DFT. To investigate the atomic‐level synergistic effect between NiCo_2_S_4_ nanosheet and graphene, DFT calculations were performed to analyze their interface interaction. Zhao et al.^[^ ^206^ ^]^ synthesized NiCo_2_S_4_ and performed a DFT study, In **Figure** **7**A, the simulated work functions of NCS (400) and graphene were found to be 4.86 and 4.57 eV, respectively. However, the work function of the NCS/graphene interface was reduced to 4.68 eV observed in Figure 7B. During the formation of NCS/graphene, owing to the higher work function of NCS, free electrons located at the graphene surface transferred to NCS to achieve equilibrium of Fermi levels, as depicted in (Figure 7C,D. Moreover, a distinct hybridization among graphene *π* orbitals, Ni 3*d* orbitals, and Co 3*d* orbitals in the vicinity of Fermi levels was observed shown in Figure 7E. This hybridization proves advantageous for the exchange of charges within the NCS‐graphene composition, contributing to the enhanced stability of the NCS/graphene electrode. Ultimately, the NCS acquires a negative charge, while graphene becomes positively charged, leading to the establishment of a Schottky electric field at the interface zone of NCS/graphene. During the charging stage, the presence of the Schottky barrier results in the temporary capture of additional electrons in the interface region, thereby augmenting the redox activity of NCS.^[^ ^207^ ^]^ This, in turn, leads to a higher capacity. For a thorough investigation into the coordination mechanism, the local electronic density of states (LDOS) at the NCS/graphene interface was examined.^[^ ^208^ ^]^

DFT calculations help in understanding the electronic properties of NiCo_2_S_4_ and its composites with graphene. By analyzing the band structure and DOS, researchers can determine how the incorporation of graphene influences the conductivity and electrochemical performance of NiCo_2_S_4_. DFT studies have shown that the presence of graphene can enhance the conductivity and the adsorption of hydroxide ions (OH^−^), which is vital for improving the performance of supercapacitors.

The unique properties of both 2D and 3D graphene‐based materials contribute to improved electrochemical performance in various applications. The different graphene morphologies play a critical role in directly influencing both the scalability of NiCo_2_S_4_/Graphene composites and their suitability for industrial applications. The choice between predominantly 2D graphene nanosheets, 3D graphene architectures (like foams or aerogels), or even crumpled graphene structures significantly impacts the ease and cost‐effectiveness of composite synthesis.^[^ ^57^, ^209^ ^]^ For instance, while 2D graphene offers high surface area for NiCo_2_S_4_ deposition, its tendency to restack can hinder scalability due to challenges in achieving uniform dispersion and consistent composite formation in large volumes. Conversely, 3D graphene frameworks, although potentially more complex to synthesize initially, can provide a robust and interconnected scaffold that facilitates uniform NiCo_2_S_4_ growth and prevents graphene aggregation, potentially leading to more scalable and reproducible production processes. Furthermore, the morphology dictates the final composite's properties, such as electrical conductivity, mechanical stability, and electrolyte accessibility.^[^ ^210^ ^]^ The composite with well‐dispersed NiCo_2_S_4_ on a conductive and porous 3D graphene network might exhibit superior electrochemical performance and durability, making it more attractive for large‐scale energy storage solutions compared to a composite with poorly dispersed NiCo_2_S_4_ on restacked 2D graphene, which might suffer from limited ion diffusion and lower cycle life. The synthesis of 3D graphene is more complex as compared to 2D graphene. Therefore, tailoring the graphene morphology is not only essential for optimizing the composite's performance but also for ensuring its cost‐effective and consistent production at an industrial scale, ultimately determining its viability for real‐world applications.

## 8 Machine Learning Analysis of NiCo_2_S_4_/Graphene Composite and its Device

ML offers powerful tools for analyzing and optimizing materials structural and electrochemical properties, which make it suitable for applications such as supercapacitors, batteries, and electrocatalysis. By leveraging ML, researchers can predict various material properties, optimize synthesis parameters, and analyze characterization data more efficiently. These models can also forecast thermal stability by predicting decomposition temperatures, enabling the development of materials that can withstand high operational temperatures. Additionally, ML can predict electrochemical performance parameters, such as capacitance, energy density, power density, and cycling stability, which are crucial for supercapacitor applications. In terms of synthesis optimization, ML algorithms can determine the optimal temperature, time, and chemical composition to achieve desired properties.^[^ ^211^, ^212^, ^213^, ^214^ ^]^

ML is transforming supercapacitor research by accelerating material discovery, optimizing performance prediction, and enabling advanced modelling. ML models like linear regression, Lasso, and artificial neural networks (ANN) analyze variables such as specific surface area, pore size, doping levels, and voltage window to predict capacitance in carbon‐based supercapacitors. ANN outperforms other methods, achieving high accuracy by learning from experimental datasets extracted from hundreds of studies. This reduces reliance on trial‐and‐error approaches and identifies optimal material configurations for enhanced energy storage. Saad et al.^[^ ^215^ ^]^ employed a ML to understand the capacitance of graphene. Ahmed Emad‐Eldeen et al.^[^ ^216^ ^]^ studied ML algorithms outperform DL for supercapacitor performance prediction. RT and XGBoost models achieve near‐perfect prediction accuracy. ML models offer efficiency and precision for simpler data relationships. ML models, such as ANN, linear regression, and Lasso regression, are employed to predict the capacitance of supercapacitors based on material properties like specific surface area, pore size, doping levels, and voltage window. These models achieve high accuracy and efficiency, enabling researchers to optimize electrode materials systematically without relying solely on experimental methods.^[^ ^217^ ^]^ This study employed ML to identify the relative importance of synthesis parameters on the specific capacitance of NiCo_2_S_4_/graphene composite materials using the algorithms. Gradient Boosting Regression (XGBoost) algorithm^[^ ^218^, ^219^ ^]^ was selected for its ability to handle nonlinear relationships, evaluate feature importance, and deliver robust predictions. XGBoost is an optimized ML model based on gradient boosting, known for its efficiency and high predictive performance. It builds an ensemble of decision trees sequentially, with each tree correcting the errors of the previous one. XGBoost is particularly effective in handling complex, nonlinear relationships, making it ideal for predicting specific capacitance, which often involves intricate interactions between experimental parameters. The model is robust to noisy data and outliers, which are common in experimental datasets, and it also offers built‐in regularization to prevent overfitting. Additionally, XGBoost provides feature importance insights, helping to identify key variables influencing capacitance. Its ability to process large datasets efficiently and its parallelization features make it well‐suited for large‐scale experiments. Overall, XGBoost's high accuracy, versatility, and ability to handle complex.^[^ ^220^, ^221^, ^222^ ^]^

The model demonstrated strong predictive performance for both NiCo_2_S_4_/graphene composite materials and devices. For composite materials, achieving a correlation coefficient of 0.60 on the test dataset and, *R* ^2^ of 0.77, and a correlation coefficient of 0.83 on the training dataset. Similarly, for NiCo_2_S_4_/graphene composite devices, the model demonstrated a correlation coefficient of 0.63 on the test dataset and *R* ^2^ of 0.92, a correlation coefficient of 0.89 on the training dataset. These results suggest that the model is effective in predicting the properties of both composite materials and devices. Feature importance analysis for composite materials^[^ ^223^ ^]^ revealed that retention, annealing time, temperature, and reaction temperature were the most influential parameters. Other factors, such as precursor and graphene concentrations, reaction conditions and electrolyte type also impacted performance but to a lesser extent. The primary factors influencing the performance of composite material devices, as determined by feature importance analysis are capacitive fading, its rate, energy density, and power density. Secondary factors, such as current density, capacitance retention, the composition and structural characteristics of the composite material, as well as the type and concentration of the electrolyte, also play a role in performance variations, though their influence is comparatively less significant. This study highlights the potential of ML to guide experimental design, deepen understanding of synthesis mechanisms, and enable the rational design of advanced energy storage materials. The dataset for this study was sourced entirely from Table 1 and 2. These datasets were processed to ensure data quality before graph preparation. Specifically, missing values (NaN) were handled using the iterative imputer technique,^[^ ^224^ ^]^ which is a robust and widely used approach in data preprocessing. Additionally, outliers were identified and removed from the datasets using established statistical criteria to further enhance the accuracy and reliability of the data. Therefore, the graphs in the manuscript are directly derived from the cleaned and pre‐processed datasets presented in the Table 1 and 2. We split the dataset into training and testing subsets to validate the model's predictive accuracy. Feature importance analysis was conducted to identify which parameters most strongly correlate with specific capacitance, providing insights into the key factors that drive electrochemical performance. This statistical approach allows for a strong and interpretable model, which can be applied to optimize material properties for high‐performance supercapacitors. Overall, ML driven analysis and optimization of NiCo_2_S_4_/graphene composite not only accelerate the development of high‐performance materials but also provide deeper insights into the underlying relationships between synthesis parameters, material structure, and properties.

**Figure 8:**
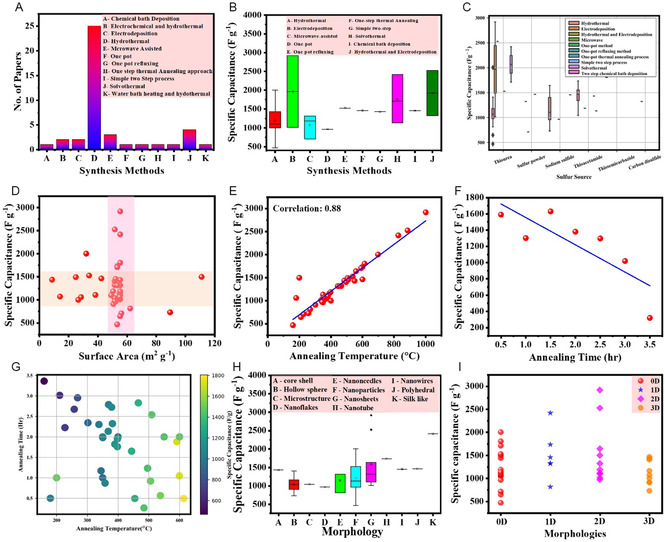
Machine learning analysis of NiCo_2_S_4_/graphene composites synthesized via different synthesis method A) Histogram of number of paper versus synthesis method. B) Box plot of specific capacitance versus synthesis methods. C) Box plot of specific capacitance versus sulfur source. D) Scatter plot of specific capacitance versus surface area. E) Scatter plot of specific capacitance versus annealing temperature. F) Scatter plot of specific capacitance versus annealing time. G) Scatter plot of annealing temperature versus annealing time. H) Scatter plot of specific capacitance versus morphology. I) Scatter plot of specific capacitance versus dimension.

Here, we studied a data of graphene composite with NiCo_2_S_4_ for ML, we have examined the experimental data of size 41 which is reported in the research article on NiCo_2_S_4_/graphene composites. The synthesis of NiCo_2_S_4_/graphene composites has been achieved through different synthesis methods as shown in **Figure** **8**A, with hydrothermal synthesis method emerging as the prevalent technique, accounting for 62.50% of the mentioned approaches. Other significant synthesis methods include microwave‐assisted method, solvothermal method, and electrodeposition, each representing 7.50% of the studies. The one‐pot method accounts for 5% of the noted syntheses. Less frequently employed techniques, each representing 2.50% of the studies, include thermal annealing approach, two‐step chemical bath deposition method, a simple two‐step process, and electrodeposition methods. This wide range of synthesis techniques illuminates the versatility and ongoing research interest in optimizing NiCo_2_S_4_/graphene composites production for several applications, with a clear preference for hydrothermal method in the current literature. The specific capacitance of NiCo_2_S_4_/graphene composites exhibited significant variation over multiple synthesis methods as shown in Figure 8B. The electrodeposition method yielded the highest maximum particular specific capacitance of 2918 F g^−1^, with a wide range of values (1321.35–2918 F g^−1^) and a mean of 2119.68 F g^−1^. The hydrothermal and electrodeposition combination synthesis method produced a consistent specific capacitance of 2526 F g^−1^. In contrast, the hydrothermal method alone resulted in lower specific capacitances, ranging from 468.51 to 2003 F g^−1^, with a mean of 1190.49 F g^−1^. The solvothermal method demonstrated moderate performance, with specific capacitances between 1133 and 2418 F g^−1^ (mean: 1753 F g^−1^). Other methods, such as microwave‐assisted synthesis (710–1320 F g^−1^), one‐pot refluxing (1526 F g^−1^), and two‐step chemical bath deposition (1454.60 F g^−1^), showed varying degrees of effectiveness. These results suggest that the electrodeposition and combined hydrothermal‐electrodeposition methods are particularly promising for achieving high specific capacitances in NiCo_2_S_4_/graphene composites. At the same time, other techniques may offer advantages in terms of process simplicity or scalability. The choice of sulfur source in the synthesis of NiCo_2_S_4_/graphene composites significantly influenced the resulting specific capacitance. The impact of morphology on the electrochemical performance of NiCo_2_S_4_/graphene composites was examined by investigating the relationship between the sulfur source and the specific capacitance, as illustrated in Figure 8C. Thiourea emerged as a versatile sulfur source, yielding a wide range of specific capacitance values over different synthesis methods. When used in the hydrothermal and electrodeposition methods, thiourea produced the highest single declared specific capacitance of 2526 F g^−1^. In solvothermal synthesis method, thiourea‐based samples exhibited a high mean specific capacitance of 2063 F g^−1^, Although with considerable variability (standard deviation: 502.05 F g^−1^). Thioacetamide also demonstrated promising results, particularly in hydrothermal synthesis method with a mean specific capacitance of 1425.78 F g^−1^ and relatively consistent performance (standard deviation: 287.09 F g^−1^). Sodium sulfide, when used in hydrothermal synthesis method, showed moderate performance (mean: 1164 F g^−1^) but with high variability (standard deviation: 346.18 F g^−1^). Remarkably, less common sulfur sources such as thiosemicarbazide and carbon disulfide yielded high single point specific capacitance values of 1804.70 and 1320 F g^−1^ respectively, Justifying further investigation. The data suggest that while thiourea and thioacetamide are promising sulfur sources for high specific capacitance of NiCo_2_S_4_/graphene composites synthesis, the interaction between sulfur source and synthesis method significantly impacts the material's electrochemical performance. The correlation between specific surface area and specific capacitance is shown in Figure 8D. Surface areas ranged from 8.78 to 111 m^2^ g^−1^ with a median of 52.20 m^2 ^g^−1^, while specific capacitances varied from 468.51 to 2918 F g^−1^. For instance, the highest specific capacitance (2918 F g^−1^) corresponded to a moderate surface area of 55.39 m^2^ g^−1^, while the largest surface area (111 m^2^ g^−1^) yielded a specific capacitance of 1498 F g^−1^. This nonlinear relationship implies that the accessibility and utilization of active sites, rather than just total surface area, play crucial roles in determining capacitive performance. Furthermore, most samples (≈75%) exhibited surface areas between 45 and 60 m^2^ g^−1^, indicating that optimizing synthesis conditions to achieve this range may be beneficial for balancing surface area and other structural factors. These results underscore the complex interaction between surface area, pore structure, and electrochemical performance in NiCo_2_S_4_/graphene composites, highlighting the need for a multifaceted approach in material design for supercapacitor applications. The annealing temperature exhibited a strong positive correlation (*r* = 0.88) with the specific capacitance of NiCo_2_S_4_/graphene composites, underscoring its critical role in determining the electrochemical performance, as shown in Figure 8E. Annealing temperatures ranged from 159.7 to 1000.9 °C, with a median of 398.1 °C. The specific capacitance increased significantly with higher annealing temperatures, ranging from 468.51 to 2918 F g^−1^. This strong relationship suggests that higher annealing temperatures generally lead to enhanced capacitive properties. The distribution of data points indicates that significant improvements in specific capacitance occur beyond 500 °C, with the majority of high‐performing samples (>1500 F g^−1^) clustered in the 600–1000 °C range. This trend can be attributed to the thermal‐induced crystallization and improved electronic conductivity of the NiCo_2_S_4_ structure, as well as better integration with the rGO sheets at elevated temperatures. Besides, the higher annealing temperatures may promote the formation of more active sites and optimize the pore structure, facilitating ion diffusion and charge storage. However, it is noteworthy that some samples annealed at lower temperatures (300–500 °C) also achieved respectable specific capacitances (1000–1500 F g^−1^), suggesting that other synthesis parameters can partially compensate for lower annealing temperatures. These outcomes emphasize the importance of carefully controlled annealing processes in optimizing the electrochemical performance of NiCo_2_S_4_/graphene composites for supercapacitor applications. The analysis of annealing time versus specific capacitance revealed a moderate negative correlation (*r* = −0.82), indicating an inverse relationship between these parameters as shown in Figure 8F. A clear downward trend is observed, with a correlation coefficient of −0.82, indicating a strong negative correlation between annealing time and specific capacitance. Annealing times ranged from 0.26 to 3.35 h, with a median of 1.94 h. The specific capacitance exhibited a general decreasing trend with longer annealing times, contrary to what might be suggestively expected. The highest specific capacitance values (>2000 F g^−1^) were predominantly observed at shorter annealing times (<2 h), while longer durations typically resulted in lower specific capacitances. In this plot, the average specific capacitance for each half hour annealing time form the plot the maximum average specific capacitance of 1620 F g^−1^ is observed for annealing time between 1 and 1.5 h, whereas the lowest average specific capacitance 250 F g^−1^ is observed in between 3 and 3.5 h. This inverse relationship suggests that prolonged annealing may lead to adverse effects such as particle agglomeration, reduced surface area, or changes in the material's crystal structure that negatively impact its electrochemical performance. The data distribution indicates that optimal annealing times for high specific capacitance (>1500 Fg^−1^) generally fall within the 0.5–1.5 h range. However, the strength of the correlation implies that other factors, such as annealing temperature or synthesis method, also play significant roles in determining the final specific capacitance. These results show the importance of carefully optimizing annealing time in conjunction with other parameters to achieve the desired electrochemical properties in NiCo_2_S_4_/graphene composites, underscoring that longer annealing durations do not necessarily translate to improved performance in supercapacitor applications. The interaction between annealing time and temperature reveals complex dynamics in their influence on the specific capacitance of NiCo_2_S_4_/graphene composites depicted in Figure 8G. Annealing temperatures ranged from 159.7 to 1000.9 °C (median: 398.1 °C), while annealing times varied from 0.26 to 6.57 h (median: 1.94 h). Notably, the highest specific capacitances (>2000 F g^−1^) were achieved with relatively high temperatures (>600 °C) and moderate annealing times (1–3 h). For instance, the maximum specific capacitance of 2918 F g^−1^ corresponded to 1000.9 °C for 2.11 h. This combination likely promotes optimal crystallization and defect healing without excessive particle growth or agglomeration. Conversely, extended annealing times (>4 h) at lower temperatures (<400 °C) generally resulted in lower specific capacitances (<1200 F g^−1^), possibly due to insufficient energy for complete structural reorganization. The data distribution indicates that the perfect balance exists in the temperature range of 600–900 °C with annealing times of 1–3 h, where 75% of samples exhibited specific capacitances exceeding 1500 F g^−1^. These observations underline the critical importance of balancing annealing time and temperature to achieve desired electrochemical properties of materials. The results suggest that a strategy of higher temperatures with moderate annealing times may be more effective in optimizing the performance of NiCo_2_S_4_/graphene composites for supercapacitor applications, likely by promoting ideal crystal structure formation and interfacial characteristics while minimizing detrimental effects associated with prolonged thermal exposure. The morphology and synthesis method of NiCo_2_S_4_/graphene composites significantly influence its specific capacitance depicted in Figure 8H. Silk‐like structures synthesized via solvothermal method exhibited the highest single stated specific capacitance of 2418 F g^−1^. Nanosheets demonstrated high specific capacitance over multiple synthesis methods, with electrodeposition method yielding a notable mean of 1965.50 F g^−1^, with high variability (standard deviation: 1347.04 F g^−1^). The hydrothermal method while widely used showed considerable variability in performance crosswise varied morphologies. For instance, nanoparticles synthesized hydrothermally displayed a wide range (468.51–2003 F g^−1^) with a mean of 1202.36 F g^−1^. Notably, unique morphologies like nanotubes and polyhedral structures, synthesized by hydrothermal and one‐step thermal annealing processes respectively, showed promising single‐point specific capacitance values above 1400 F g^−1^. The combination of hydrothermal and electrodeposition methods for nanosheets yielded high specific capacitance (mean: 1923.66 F g^−1^), suggesting synergistic benefits. Overall, while certain morphologies like silk‐like structures and nanosheets tend to exhibit higher specific capacitance values, the synthesis method plays a crucial role in determining the final electrochemical performance of NiCo_2_S_4_/graphene composites. Figure 8I illustrate the relationship between specific capacitance and the dimensionality of NiCo_2_S_4_/graphene composites structures transversely multiple synthesis methods. The hydrothermal method demonstrated remarkable versatility, producing structures throughout all dimensions with varying specific capacitances. For 0D structures, hydrothermal synthesis method yielded a wide range of specific capacitances (468.51–2003 F g^−1^, mean: 1209.03 F g^−1^), highlighting its potential for fine‐tuning nanoparticle properties. The solvothermal method showed promise for both 0D (mean: 1420.50 F g^−1^) and 1D structures, with the latter achieving the highest single specific capacitance value of 2418 F g^−1^ for silk‐like morphologies. In the 2D structures, electrodeposition and combined hydrothermal‐electrodeposition methods are outstanding, producing nanosheets with high mean specific capacitances of 1965.50 and 1923.66 F g^−1^, respectively, even though with significant variability. The 3D structures, while generally showing lower capacitances compared to lower‐dimensional counterparts, demonstrated consistent performance beyond diverse synthesis methods, with the one‐step thermal annealing process achieving 1463 F g^−1^ for polyhedral structures. Notably, the microwave‐assisted method demonstrated a notable pattern of increasing specific capacitance with dimensionality (0D ‐ 710 F g^−1^, 1D ‐ 1320 F g^−1^, 2D ‐ 1186 F g^−1^). These results emphasize the complex interaction between dimensionality, synthesis methods, and resulting specific capacitance in NiCo_2_S_4_/graphene composites. While lower‐dimensional structures generally exhibited higher specific capacitances, likely due to their increased surface area and efficient electron transport pathways, the synthesis methods played a crucial role in determining the final electrochemical performance, with solvothermal and electrodeposition techniques showing promise for achieving high specific capacitance values. To identify the exact evaluation and relation between specific capacitance and other parameter, we considered to a hydrothermal method for ML.

**Figure 9:**
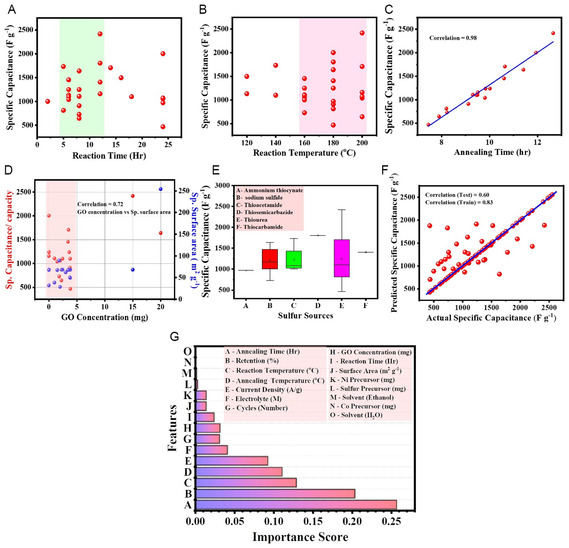
Machine learning analysis of supercapacitor behavior of hydrothermally synthesized NiCo_2_S_4_/graphene composites. A) Scatter plot of specific capacitance versus reaction time. B) Scatter plot of specific capacitance versus reaction temperature. C) Scatter plot of specific capacitance versus annealing time. D) Scatter plot of specific capacitance versus graphene concentration. E) Scatter plot of specific capacitance versus sulfur source. F) Scattered plot of experimental specific capacitance (F g^−1^) versus predicted specific capacitance (F g^−1^) by gradient boosting tree machine learning model of NiCo_2_S_4_ and NiCo_2_S_4_/rGO materials. G) Histogram of important features with respect to specific capacitance (F g^−1^) calculated with gradient boosting tree machine learning model of NiCo_2_S_4_ and NiCo_2_S_4_/rGO materials.

Here we are using a specific synthesis method hydrothermal for further ML analysis for NiCo_2_S_4_/graphene composite. The analysis of specific capacitance as a function of reaction time in the dataset highlights a complex relationship for NiCo_2_S_4_/graphene composites illustrated in **Figure** **9**A. Specific capacitance values span a broad range from 468.51 to 2918 F g^−1^, corresponding to reaction times varying from 1 to 24 h. A general trend emerges implying that prolonged reaction times often correlate with enhanced specific capacitance, though this relationship is not strictly linear or uniformly consistent over all data points. Notably, some of the highest capacitance values (exceeding 2000 F g^−1^) are observed for reaction times of 12 and 24 h, indicating that extended synthesis time can promote the development of highly capacitive structures. However, the data also present intriguing exceptions, with some materials achieving remarkably high specific capacitance (below 1500 F g^−1^) at shorter reaction times of 5–8 h. This observation shows that optimized synthesis conditions can potentially yield high‐performance materials with reduced processing times, a finding of significant interest for scalable manufacturing processes. The distribution of data points throughout the capacitance‐reaction time space exhibits considerable scatter, underscoring the multifaceted nature of the relationship. Materials synthesized with moderate reaction times (6–12 h) frequently display capacitance values in the range of 1000–1500 F g^−1^, potentially representing a practical compromise between processing duration and electrochemical performance. The relationship between specific capacitance and reaction temperature in the dataset unveils significant insights into the synthesis‐structure‐property relationships of the electrochemical materials under investigation depicted in Figure 9B. Specific capacitance values range from 468.51 to 2918 F g^−1^, corresponding to reaction temperatures spanning from 95 to 200 °C. This broad range of conditions and outcomes underscores the critical role of thermal energy in determining the final electrochemical properties of the synthesized materials. A notable trend emerges where in higher reaction temperatures generally correlate with increased capacitance values, although this relationship is not strictly linear. The data recommend the existence of an optimal temperature range, ≈160–200 °C, where many of the highest capacitance values (above 1500 F g^−1^) are observed. This optimal range likely represents a balance between enhanced reaction kinetics and the preservation of desirable structural features conducive to high capacitance. Interestingly, the dataset exhibits several outliers that achieve exceptional capacitance (above 2000 F g^−1^) at both moderate (180 °C) and elevated (200 °C) temperatures. These data points are of particular interest as they may indicate specific synthesis conditions or precursor compositions that yield superior electrochemical performance. Conversely, materials synthesized at lower temperatures (below 140 °C) tend to exhibit lower capacitance values, indicating insufficient thermal energy for the formation of optimal electrochemically active structures. The analysis of the relationship between specific capacitance and annealing time as shown in Figure 9C shows intriguing trends with significant implications for the optimization of electrochemical energy storage materials. The dataset exhibits specific capacitance values ranging from 468.51 to 2918 F g^−1^, corresponding to annealing times varying from ≈3 to 13 h. The observed positive correlation coefficient of 0.98 between specific capacitance and annealing time indicates a moderate to strong positive relationship, implying that extended annealing periods generally contribute to enhanced capacitance. The data discovers that many of the highest specific capacitance values (above 1500 F g^−1^) are associated with annealing times exceeding 3.5 h, underscoring the importance of sufficient thermal treatment in achieving desirable electrochemical properties. Notable outliers in the dataset, such as materials achieving high capacitance (>1500 F g^−1^) with relatively short annealing times (<10 h), merit further investigation. These exceptions may represent unique material compositions or synthesis conditions that enable rapid optimization of electrochemical properties, potentially offering pathways for more time‐efficient manufacturing processes. The plot GO concentration vs specific capacitance and GO concentration vs specific surface area are shown in Figure 9D. The plot shows the higher specific capacitance (1500–2000 F g^−1^) for GO concentration up to 4.5 mg and the specific surface area up to 100 m^2^ g^−1^ up to 2.5 mg GO concentration. But there are some exceptions can be observed for the GO concentration of above 15 mg. Furthermore, the GO concentration exhibited a positive correlation of with the specific capacitance and a strong positive correlation of 0.72 with the specific surface area, implying that increasing the GO content can effectively increase the specific surface area of the nanocomposites, thereby enhancing their capacitive performance illustrates in Figure 9D. Due to limited data, a clear trend is not noticeable. However, some exceptions can be observed, particularly for GO concentrations exceeding 5 mg. The analysis of specific capacitance values for NiCo_2_S_4_/graphene composites materials synthesized using numerous sulfur sources via hydrothermal method exhibits significant insights into the impact of precursor chemistry on electrochemical performance as shown in Figure 9E. Thiourea emerged as the sulfur source yielding the widest range of specific capacitance values, spanning from 468.51 F g^−1^ to an impressive 2918 F g^−1^, with a mean of 1384.39 F g^−1^ and a substantial standard deviation of 638.49 F g^−1^. This broad distribution offers that thiourea‐based synthesis offers high potential for capacitance optimization but also requires careful control of reaction parameters. Sodium sulfide and thioacetamide displayed more consistent performance, with narrower ranges and lower standard deviations. Sodium sulfide produced materials with capacitances ranging from 729 to 1640 F g^−1^ (mean: 1175.20 F g^−1^, SD: 321.40 F g^−1^), while thioacetamide yielded a range of 1040.60 to 1733 F g^−1^ (mean: 1350.59 F g^−1^, SD: 241.85 F g^−1^). The higher mean and lower variability of thioacetamide‐derived materials display it may offer a more reliable route to high‐performance electrodes. Interestingly, thiosemicarbazide and carbon disulfide each showed single data points with high specific capacitances of 1804.70  F g^−1^ and 1320 F g^−1^, respectively. While these results are promising, the lack of multiple data points precludes statistical analysis and necessitates further investigation to determine reproducibility and optimize conditions. Sulfur powder demonstrated moderate performance with a mean specific capacitance of 1164.78 F g^−1^ and a range from 710 to 1463 F g^−1^. The standard deviation of 400.17 F g^−1^ indicates significant variability, indicating that synthesis conditions play a crucial role in determining the final electrode performance when using elemental sulfur as a precursor. We identified the predicted specific capacitance equation from actual capacitance value by using ML data as shown in Equation (5)

(5) $${\text{Pre}}_{\text{Sc}}=0.76\times {\text{Act}}_{\text{Sc}}+339.19$$

where ${\text{Pre}}_{\text{Sc}}$ is the predicted specific capacitance and ${\text{Act}}_{\text{Sc}}$ is the actual specific capacitance. Equation (5) is derived from the linear regression of predicted specific capacitance versus actual capacitance. The equation represents the best‐fit line, where the slope (0.76) and the y‐intercept (339.19) are the result of the least squares fitting process. The slope reflects the rate of change in actual capacitance with respect to predicted capacitance, while the y‐intercept represents the value of the actual capacitance when the predicted capacitance is zero. These parameters were obtained by performing a linear fit on the data points corresponding to the predicted and actual capacitance values. Specifically, the annealing time emerged as the most crucial factor, suggesting that the duration of the annealing process plays a pivotal role in determining the specific capacitance of the nanocomposites. This could be attributed to the potential enhancement of crystallinity, conductivity, and structural integrity during prolonged annealing, thereby improving the electrochemical performance. The cyclic retention, which measures the capacity retention over multiple charge–discharge cycles, was the second most important feature. This results the significance of achieving stable and durable electrochemical performance, as nanocomposites with better cyclic stability are likely to exhibit higher specific capacitance values. Other influential features included the reaction temperature, annealing temperature, and current density, indicating that the synthesis conditions and electrochemical testing parameters have a considerable impact on the specific capacitance of the nanocomposites. Interestingly, the precursor concentrations (Ni precursor and sulfur precursor) and the GO concentration were found to have relatively lower importance compared to the previously mentioned features. This suggests that while the precursor ratios and GO content play a role in determining the nanocomposite properties, their influence on the specific capacitance may be overshadowed by other factors, such as the annealing conditions and cyclic stability. For model validation, we calculated the *R* ^2^ value, which measures the proportion of variance in the dependent variable that is explained by the independent variables in the model. In our case, the *R* ^2^ value was found to be 0.92, indicating that the model explains 98% of the variance in the specific capacitance predictions. To further validate the predictive performance of the ML model, a plot comparing the actual and predicted specific capacitance values Figure 9F for the testing dataset (25% of the data) was generated. The correlation coefficient of 0.60 between the actual and predicted values indicates a moderate to strong relationship, suggesting that the model can provide reasonably accurate predictions of the specific capacitance based on the input features. The feature importance analysis revealed valuable insights into the relative influence of different synthesis parameters and material properties on the specific capacitance of NiCo_2_S_4_/graphene composites. On the basis of significant features data, we observed the most significant feature influencing the specific capacitance is an annealing time, followed by the cyclic retention, reaction temperature, annealing temperature, and current density as shown in Figure 9G. In this analysis, we prioritized the discussion of parameters that exhibited a strong correlation (≥50%) with specific capacitance. This approach was adopted to identify the key factors directly influencing the electrochemical performance of NiCo_2_S_4_/graphene composite materials. Parameters that were excluded from the analysis include those that were either poorly reported in the majority of the studies, which could lead to abundances in the dataset. For instance, while particle size and electrode configuration may also affect capacitance, they were not included due to insufficient consistency in reporting across the dataset. Furthermore, we excluded parameters that were not directly related to the synthesis and material characterization processes, as our focus was on understanding how these factors influence electrochemical performance. While cycling parameters are undoubtedly significant in overall electrochemical performance, their correlation with specific capacitance was found to be less pronounced. Among various alkaline electrolytes, potassium hydroxide (KOH) was selected due to its superior ionic conductivity of 0.6 S cm^−^^1^ at 25 °C for an optimal concentration of 6 m. Alkaline electrolytes being mildly acidic exhibit higher ionic conductivity compared to neutral electrolytes.^[^ ^225^ ^]^ Consequently, KOH was chosen as the sole electrolyte for this investigation. As the electrolyte concentration variations within the dataset were minimal, the effects of electrolyte type and concentration. Therefore, there is scope to investigate effect of electrolyte and their concentration on specific capacitance. Overall, this analysis underlines the critical role of synthesis parameters, material properties, surface area, and morphology in determining the specific capacitance of NiCo_2_S_4_/graphene composites. Surface area particularly important because they govern the material's ability to interact with ions, thereby affecting capacitance. Additionally, capacitance retention is a key factor as it reflects how well the material maintains its capacitance over repeated charge–discharge cycles. The choice of electrolyte and current density conditions can also influence specific capacitance by altering ion mobility and the material's response during electrochemical testing. The feature importance analysis provides valuable insights for optimizing the synthesis process and tailoring the nanocomposite properties to achieve enhanced electrochemical performance for energy storage applications.

In conclusion, our ML study aimed to evaluate the relative importance of precursors and reaction conditions in predicting specific capacitance with a particular focus on leveraging the Shapley Additive explanations (SHAP) tool^[^ ^226^, ^227^ ^]^ for explainable analysis. The SHAP results revealed that the sulfur source, molar concentration of precursors, reaction conditions, and annealing parameters were the most influential factors. The sulfur source significantly impacted the material's conductivity and active site availability. This aligns with observations that sulfur‐based functional groups depending on their bonding environment can enhance charge storage and electron transfer. For instance, using elemental sulfur versus thiourea as the sulfur source yielded differing levels of porosity and surface area, which directly correlated to variations in capacitance. Similarly, the molar concentration of metal precursors influenced the stoichiometry and crystalline phase of the resulting nanomaterial. Higher concentrations favored the formation of well‐crystallized structures which improved electrical conductivity but sometimes compromised the surface area due to particle agglomeration. This duality highlights the need for optimizing precursor ratios to balance these properties. The reaction conditions such as temperature and solvent type were found to affect particle morphology and defect density. For example, higher temperatures promoted the formation of nanostructures with enhanced interconnectivity which improved ion transport pathways. However, excessive annealing temperatures led to grain growth and reduced surface area, negatively affecting the capacitance. The annealing parameters were crucial for phase stabilization and defect engineering. Annealing at moderate temperatures ensured the removal of unreacted precursors and residual stresses, resulting in defect‐rich materials conducive to charge storage. However, overly aggressive annealing reduced the electrochemical active sites, underscoring the importance of controlled thermal treatment. These observations highlight the complex interaction between synthesis parameters and their impact on material properties. Our results emphasize that a tailored approach accounting for these interdependencies is essential for optimizing electrochemical performance. By correlating SHAP‐derived insights with experimental outcomes, we provide a more comprehensive understanding of the underlying mechanisms driving specific capacitance.

**Figure 10:**
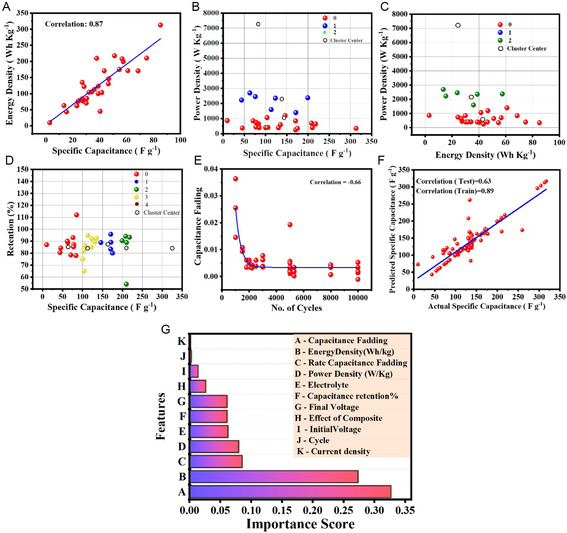
Machine learning analysis of supercapacitor behavior of NiCo_2_S_4_/graphene composite device; scatter plot of A) specific capacitance versus energy density, B) specific capacitance versus power density, C) energy density versus power density, D) capacitance retention versus specific capacitance. E) Number of cycle versus capacitance fading. F) Scattered plot of experimental specific capacitance (F g^−1^) versus predicted specific capacitance (F g^−1^) by gradient boosting tree machine learning model of NiCo_2_S_4_ and NiCo_2_S_4_/rGO device. G) Histogram of important features with respect to specific capacitance (F g^−1^) calculated with gradient boosting tree machine learning model of NiCo_2_S_4_ and NiCo_2_S_4_/rGO device.

Here, the fabricated NiCo_2_S_4_/graphene composite devices were analyzed using ML. ML supports us in fabricating desirable devices. The graphene composite with NiCo_2_S_4_ dataset contains 40 data points. The initial voltage (0 V) in all cases and final voltage ranges from 0.8 to 1.8 V, with a mean of 1.51 V and a standard deviation of 0.24 V. KOH is used as an electrolyte with concentrations ranging from 1 to 6 m, representing different electrolyte compositions. The energy density has a mean of 39.28 Wh kg^−1^ and a standard deviation of 18.18 Wh kg^−1^, ranging from 2.9 to 85.1 Wh kg^−1^. The power density has a mean of 2356.49 W kg^−1^ and a standard deviation of 4769.37 W kg^−1^, with a wide range from 254.3 to 22,000 W kg^−1^. The specific capacitance has a mean of 126.54 F g^−1^ and a standard deviation of 65.68 F g^−1^, ranging from 9.86 to 313 F g^−1^. The multivariate analysis reveals interesting correlations among the parameters. A stronger positive correlation of 0.87 between energy density and specific capacitance depicted in **Figure** **10**A, featuring the synergistic effect of incorporating rGO in enhancing capacitive performance. In Figure 10B, we explore three clusters in the specific capacitance with power density, explaining 94.49% of the data variance. This analysis offers insights into the electrochemical performance of NiCo_2_S_4_/graphene composite. The first cluster (124.5734 F g^−1^, 2103.7551 W kg^−1^) represents samples with a balanced combination of moderate capacitance and high‐power density, suggesting efficient charge storage and rapid charge–discharge capabilities. The second cluster, with lower capacitance but exceptionally high‐power density (62 F g^−1^, 7418 W kg^−1^), highlights samples with superior rate capability due to highly conductive pathways provided by the rGO component, although with reduced capacitance. The largest cluster (130.4632 F g^−1^, 590.9367 W kg^−1^) shows the highest average capacitance but moderate power density, likely representing NiCo_2_S_4_/graphene composite with a higher proportion of NiCo_2_S_4_, leading to increased charge storage capacity but somewhat lower power density. Figure 10C illustrates three clusters in the energy density and power density space, explaining 94.68% of the data variance. This analysis provides crucial insights into the energy storage and power delivery capabilities of NiCo_2_S_4_/graphene composite. The first cluster (23 Wh kg^−1^, 7418 W kg^−1^) represents samples with moderate energy density but exceptionally high‐power density, likely due to optimized rGO content and distribution, facilitating rapid electron transport and ion diffusion. The second cluster (34.6826 Wh kg^−1^, 2103.7551 W kg^−1^) shows a balanced combination of energy density and power density, suggesting well‐tuned NiCo_2_S_4_ to rGO ratios. The third and largest cluster (42.0386 Wh kg^−1^, 590.9367 W kg^−1^) has the highest average energy density but moderate power density, indicating NiCo_2_S_4_/graphene composite with a higher proportion of NiCo_2_S_4_, suitable for applications prioritizing energy density over rapid charge–discharge capabilities. Finally, Figure 10D shows five clusters in the specific capacitance‐retention space, explaining 92.73% of the data variance. This analysis reveals intriguing patterns in the performance characteristics of the NiCo_2_S_4_/graphene composite. The cluster with the highest specific capacitance (313 F g^−1^, 83.1%) represents an exceptional NiCo_2_S_4_/graphene composite that combines high charge storage capacity with good cycling stability. Another cluster, with the highest average retention (74.4412 F g^−1^, 89.0931%), shows lower specific capacitance, suggesting that some NiCo_2_S_4_/graphene compositions may prioritize long‐term stability over initial capacitance. The largest cluster (192.5356 F g^−1^, 85.4467%) demonstrates a balance between high specific capacitance and very good retention, likely representing an optimal NiCo_2_S_4_/graphene composition offering both high performance and durability. Specific capacitance and capacitance fading indicate that materials with higher specific capacitance tend to experience more capacity fading over cycles, which could be due to the increased strain on the electrode material during charging and discharging processes. The positive correlations of 0.47 between rate of capacitance fading and current density, this is an important consideration for designing high‐power energy storage devices with good cycle stability. These correlations provide valuable insights into the interaction between different parameters and can guide the development of high‐performance electrochemical energy storage materials and devices. Further analysis and experimental validation may be required to establish causal relationships and develop predictive models. The rate of capacitance fading over cycle fitting process yielded the parameter values *A* = 0.30, *B* = 379.39, *C* = 0.0034. The rate of capacitance fading per 1000 cycles was estimated to be 22.13 F g^−1^, with a correlation coefficient of ‐0.66 between the rate of capacitance fading and cycle number illustrated in Figure 10E. This negative correlation suggests that the rate of capacitance fading decreases as the cycle number increases, potentially due to initial rapid degradation followed by stabilization of the electrode material. The lower rate of capacitance fading for the NiCo_2_S_4_/graphene composites indicates improved cyclic stability compared to the NiCo_2_S_4_, likely attributable to the incorporation of rGO. The model provides a useful tool for predicting the remaining capacity or energy density of electrochemical energy storage devices after a certain number of cycles, although further validation and investigation into the underlying degradation mechanisms are recommended. The ML analysis of combine dataset of NiCo_2_S_4_ and NiCo_2_S_4_/graphene composites provide the following information, the final voltages varied from 1.0 to 1.8 V, with 1.6 V as the predominant value. ML analysis using a *t*‐test and ANOVA revealed no significant differences in the initial voltage (t‐statistic = 0.25, *p*‐value = 0.80) and final voltage (t‐statistic = −0.06, *p*‐value = 0.94) between the NiCo_2_S_4_ and NiCo_2_S_4_/rGO samples. The energy density, a critical parameter for energy storage applications, ranged from 2.9 to 76.7 Wh kg^−1^, with a mean value of 38.9 Wh kg^−1^. Similarly, the power density, which governs the rate capability, spanned from 7.17 to 7418 W kg^−1^, with an average of 906.54 W kg^−1^. The *t*‐test and ANOVA results indicated no significant difference in energy density (*t*‐statistic = 0.64, *p*‐value = 0.52) but a statistically significant difference in power density (*t*‐statistic = −2.41, *p*‐value = 0.017) between the two sample groups. The specific capacitance, a measure of the capacitive performance, exhibited values ranging from 9.861 to 259.6 F g^−1^, with a mean of 125.26 F g^−1^. Notably, a moderate positive correlation of 0.47 was observed between energy density and specific capacitance, suggesting that higher specific capacitance values tend to contribute to enhanced energy storage capabilities. However, the *t*‐test and ANOVA results showed no significant difference in specific capacitance (*t*‐statistic = 0.64, *p*‐value = 0.51) between the NiCo_2_S_4_ and NiCo_2_S_4_/graphene composites. The applied current densities varied from 0.01 to 6.902 A g^−1^, with an average of 1.10 A g^−1^. A moderate positive correlation of 0.54 was found between power density and current density, which is expected as higher current densities enable faster charge/discharge rates and, consequently, higher power delivery. The ML analysis revealed that no significant difference in current density (*t*‐statistic = 1.47, *p*‐value = 0.14) between the two sample groups. The capacitance retention percentage, a crucial indicator of cyclic stability, ranged from 46.4% to 100%, with a mean value of 84.7%. The *t*‐test and ANOVA results showed a marginally significant difference in capacitance retention percentage (*t*‐statistic = −1.9, *p*‐value = 0.0558) between the NiCo_2_S_4_ and NiCo_2_S_4_/graphene composites, suggesting potential differences in cyclic stability. The ML analysis aimed to identify the most influential parameters affecting the specific capacitance of the NiCo_2_S_4_/graphene composites. It is worth mentioning that the moderate correlations between the actual and predicted specific capacitance values (0.63 for testing data and 0.83 for training data indicated in Figure 10F suggest a reasonable predictive capability of the model. However, due to the limited amount of data available, the feature importance analysis should be considered a rough estimation, and further experiments and data collection may be required to refine the understanding of the most crucial input parameters influencing the specific capacitance. Among the input parameters, the feature importance analysis revealed that the electrolyte composition had a significant impact on the specific capacitance. Interestingly, the initial voltage (0.014 V) and final voltage (0.061 V) also exhibited moderate influence, suggesting that the voltage window plays a role in determining the specific capacitance. Lastly, the current density was identified as a relatively less important input factor affecting the specific capacitance. On the other hand, the output parameters, such as capacitance fading (0.3276), energy density (0.2743), rate capacitance fading (0.085), and power density (0.080), were found to be influential features, indicating their strong correlation with the specific capacitance as shown in Figure 10G. However, it is important to note that these output parameters are derived from the experiments and are not directly controllable input variables.

ML accelerates the discovery and design of materials by analyzing their atomic and electronic structures. For example, ML methods have been used to identify defects in materials like aluminum and polyethylene, which impact electronic efficiency. This allows researchers to design superior materials faster than conventional quantum mechanics‐based techniques. ML models such as ANN, random forest (RF), and XGBoost are used to forecast the CV behavior of supercapacitors and optimize their energy density, power density, and utilization. These predictions align closely with experimental results, demonstrating the reliability of data‐driven approaches in improving supercapacitor performance. Predicting the remaining useful life of supercapacitors is crucial for applications in transportation and renewable energy systems.^[^ ^228^, ^229^ ^]^ ML techniques like recurrent neural networks (RNN) and long short‐term memory (LSTM) networks are applied to time‐series data to forecast lifecycle performance accurately. This helps in planning maintenance schedules and preventing system failures. ANN‐based models simulate the electrical behavior of supercapacitors under varying conditions like temperature and current. These models provide insights into charge–discharge cycles and validate experimental results effectively, aiding in applications such as transportation and power electronics.^[^ ^230^, ^231^ ^]^ ML‐driven advancements have been applied to IoT applications by optimizing supercapacitor designs for low‐power systems. Additionally, novel approaches like integrating quantum dots with supercapacitors are explored using AI‐based analytical formulations. ML study helps to predict the morphology of the sample before the synthesis which reduces the cost of the research.^[^ ^232^, ^233^ ^]^ On the basis precursor concentration and volume of solvent, we can easily identified the probability of the morphology of the material. With the help of ML, we can easily synthesize a desired morphology through an experimental process.^[^ ^234^, ^235^, ^236^ ^]^ The ML study helps to find the predict the specific capacitance of the material because of the surface area, morphology, and other parameter, which helps to reduce the experimental work. With the help of ML, we can easily scale the experimental parameter to the industrial level. One of the major challenges in supercapacitor technology is balancing energy density and power density. Traditional supercapacitors exhibit high‐power density but often suffer from limited energy storage capacity. The findings highlight how tuning synthesis methods, solvent selection, and surfactant use can optimize NiCo_2_S_4_/graphene morphology, leading to improved electrochemical performance. The Kirkendall effect and Ostwald ripening contribute to the formation of hollow and mesoporous NiCo_2_S_4_ structures, increasing surface area and ion diffusion pathways. This directly enhances charge storage capacity. Exploring novel 2D nanostructured morphologies to maximize surface area and charge storage capabilities. Investigate alternative synthesis techniques beyond hydrothermal method to enhance material performance. Optimizing the NiCo_2_S_4_/graphene interface to further improve electrical conductivity and mechanical stability.

## 9 Conclusion and Outlook

NiCo_2_S_4_ materials are favored for their affordability, oxidation activity, and diverse crystal phases. NiCo_2_S_4_ is particularly promising due to sulfur low electronegativity, which enhances structural stability, and the higher conductivity of sulfides compared to oxides, leading to superior electrochemical performance. In this review, an overview of recent advancements in the synthesis methods, growth mechanisms, morphology control techniques, surfactant and solvent roles, and electrochemical properties of NiCo_2_S_4_/graphene composites is reviewed. Simultaneously, we outline the strengths and weaknesses of existing synthesis methods for NiCo_2_S_4_/graphene composites, including the hydrothermal/solvothermal, electrodeposition, microwave‐assisted, and template methods. The synthesis of NiCo_2_S_4_/graphene composites has predominantly focused on the hydrothermal method. This method ensures controlled morphology and enhanced crystallinity of NiCo_2_S_4_, optimizing its electrochemical performance for supercapacitor applications over other methods. The controlled sulfurization of NiCo_2_S_4_ nanostructures, by adjusting sulfur source and sulfurization duration to manipulate sulfur vacancy concentration and impurities, significantly impacts the supercapacitive performance. Ostwald ripening plays a crucial role in the synthesis of NiCo_2_S_4_/graphene composites by facilitating the growth of uniform and well‐defined nanostructures. The utilization of the Kirkendall effect proves invaluable in the synthesis and morphology control of NiCo_2_S_4_/graphene composites. The 1D or 2D nanostructures are typically employed as building blocks, resulting in the creation of 3D core–shell structures. However, several challenges remain for NiCo_2_S_4_‐based supercapacitors, including lower conductivity and cyclic stability compared to carbon materials. Therefore, integrating carbon materials like graphene composites can enhance both the conductivity and cyclic stability of NiCo_2_S_4_. It is important to note that the ML analysis showed a 3D NiCo_2_S_4_ nanomaterials exhibited a higher surface area and porous nature, also it was observed that 3D nanostructured electrode materials, such as hierarchical, hollow, and core–shell structures, offer larger specific surface areas, enhancing their electrochemical performance. 3D graphene, with its augmented surface area and porosity relative to 2D graphene, emerges as an optimal candidate for energy storage applications. In conclusion, the optimization of hydrothermal synthesis parameters, including precursor concentration, reaction time, reaction temperature, annealing conditions, and morphology, is a critical factor in enhancing the electrochemical performance of NiCo_2_S_4_/graphene composites supercapacitors, as evidenced by the experimental results.

## 10 Future Perspective

1) Fabricating NiCo_2_S_4_ with customized nanostructures typically involves a two‐step process: first forming nanostructured oxides, then sulfurizing simultaneously. However, sulfurization induces defects and lattice distortions that particularly reduce electron diffusion length. Thus, developing a simple one‐step synthesis method for adjustable nanostructured NiCo_2_S_4_ remains a significant challenge; 2) the introduction of sulfur vacancies in NiCo_2_S_4_ enhances active sites, but it impacts on cyclic stability. The mechanism of their influence remains unclear; therefore, there is scope to balancing defect concentration is crucial for optimizing electrode materials; 3) during a sulfurization process, a large number of voids are created. Which provide an active site but this technique promotes the formation of a more condensed and resilient structure. Thus, there is risk of collapse during the charge/discharge phases in supercapacitor operation. So, there is a need to enhance the structural stability of NiCo_2_S_4_/graphene composite for supercapacitors and the researchers need to utilize a one‐step synthesis method; 4) mostly, we observed that hydrothermal and solvothermal methods are employed to synthesize NiCo_2_S_4_ graphene composite nanostructures. So, there is huge scope to synthesize NiCo_2_S_4_ graphene composites using different chemical methods other than hydrothermal and solvothermal methods and investigate supercapacitor performance; and 5) graphene sheets tend to stack on top of each other due to π‐π interactions, which can limit the accessible surface area and affect the overall performance of the electrode material. This stacking reduces the effective surface area available for charge storage, thereby reducing the capacitance of the electrode. Moreover, the stacked graphene layers can also hinder the diffusion of electrolyte ions into the electrode material, further impacting its electrochemical performance. There is opportunity to solve this problem by further development of functionalized or doped graphene derivatives; 6) developing 0D NiCo_2_S_4_ nanoparticles is challenging due to the necessity of a nucleation surface for particle growth. There is scope to explore chemical synthesis methods other than hydrothermal for developing 0D NiCo_2_S_4_ nanoparticles on 2D graphene sheets; 7) the specific capacitance of the electrode material can be compromised if there is insufficient contact between NiCo_2_S_4_ and GO. This inadequate interfacial contact can arise due to poor dispersion of NiCo_2_S_4_ on the surface of GO due to the presence of insulating impurities between them. So, there is a chance to enhance specific capacitance, ensure uniform dispersion of NiCo_2_S_4_ on GO via methods like ultrasonication, or use LBL assembly techniques to optimize interfacial contact; 8) there is scope to develop a core–shell‐like structure, which provide a higher material stability during charge discharge process; 9) there is a need to exploring electrolyte such as ionic liquids, organic solutions, and redox species integration into aqueous systems aims to boost electrochemical performance of NiCo_2_S_4_ and GO‐based nanocomposite; and 10) future research can use predictive algorithms to streamline synthesis protocols for tailored electrochemical properties. Also, there is scope to explore any other parameters related to the materials structural and electrochemical properties of NiCo_2_S_4_‐based composites.

Constructing 3D NiCo_2_S_4_‐based composites with a controlled pore size distribution is crucial for developing electrode materials. Utilizing 3D conducting materials as templates and incorporating electroactive materials such as carbon‐based materials, CPs, TMOs, and TMSs within these structures is a proven method for creating efficient electrodes. Further research should focus on developing specific synthesis techniques for fabricating nanostructures to advance this field.

## Conflict of Interest

The authors declare no conflict of interest.
